# Single amino acid substitution in Hendra virus attachment glycoprotein induces cross-neutralizing antibodies against Nipah virus

**DOI:** 10.1038/s41392-025-02370-0

**Published:** 2025-08-29

**Authors:** Yaohui Li, Xiaoyan Huang, Ruihua Li, Xiaodong Zai, Yilong Yang, Yue Zhang, Zhang Zhang, Jun Zhang, Junjie Xu, Wei Chen

**Affiliations:** Laboratory of Advanced Biotechnology, Beijing Institute of Biotechnology, Beijing, China

**Keywords:** Vaccines, Structural biology

## Abstract

Nipah virus (NiV) and Hendra virus (HeV) are highly pathogenic henipaviruses within the Paramyxoviridae family, causing severe respiratory and neurological diseases in humans and animals with fatality rates up to 75%, and no licensed human vaccines or therapeutics. In this study, we identified a unique vulnerable epitope on the NiV attachment glycoprotein (G) recognized by the potent neutralizing antibody 14F8, which targets a receptor-binding site and neutralizes NiV effectively. Using the 2.8 Å crystal structure of the 14F8 Fab–NiV-G complex as a guide, we reconstructed this epitope on HeV-G via a single amino acid substitution (S586N), creating the HeV-G_S586N_ mutant. Immunization with HeV-G_S586N_ in BALB/c mice and cynomolgus monkeys elicited robust, broadly neutralizing antibody responses against both NiV and HeV, achieving higher NiV-neutralizing titers post-prime compared to wild-type HeV-G, as confirmed by pseudovirus and live-virus assays. Crystal structures of HeV-G_S586N_ (3.3 Å) and its 14F8 complex (3.2 Å) showed the S586N substitution induced a 9 Å conformational rearrangement in β-propeller blade 6, reshaping the molecular skeleton and solvent-accessible surface without direct N586–14F8 interaction, thus mimicking the NiV epitope. These findings position HeV-G_S586N_ as a promising broad-spectrum antigen for henipavirus prevention and demonstrate the value of structure-guided epitope reconstruction in universal vaccine design for emerging viral threats.

## Introduction

*Henipavirus* (HNV) is a genus of single-stranded negative-sense RNA viruses, among which the Hendra virus (HeV) and Nipah virus (NiV) can cause severe respiratory syndromes and encephalitis in humans.^1^ They are classified as virulent pathogens by the World Health Organization, but priority research is needed due to their significant threat to public health and the lack of specific antiviral treatments. HeV was identified for the first time in 1994 after an outbreak in the town of Hendra, Australia,^2^ where it caused fatal pneumonia in horses and humans. Notably, in 2022, a novel variant of HeV-2 was detected in a horse located in Queensland, Australia.^3,4^ This new variant exhibited genetic differences from the original strain, raising concerns about its potential for increased transmissibility and virulence. NiV was first reported during outbreaks in 1998 in Malaysia and Singapore and can be subdivided into Malaysian and Bangladeshi strains.^5–7^ The Malaysian strain is often associated with porcine-to-human transmission, while the Bangladeshi strain has a more direct bat-to-human transmission route, highlighting the complex epidemiology of NiV. This emergent genetic and geographic variability among HNVs underscores the urgent need for the development of universal vaccines able to provide broad coverage against various outbreaks. Current prevention strategies, primarily dependent on surveillance and biosecurity measures, demonstrate limited efficacy in containing sporadic and sudden outbreaks. Therefore, the development of a universal vaccine has become an imperative to safeguard global public health against these highly pathogenic viruses.

Despite decades of research, there are no licensed vaccines against HeV or NiV for humans, although there are over 40 vaccine candidates are in various stages of development. The majority of candidate vaccines use the attachment glycoprotein G as an antigen, which recognizes the receptors Ephrin B2 or Ephrin B3 on the host cell.^8–27^ The protective effects of HNV-derived recombinant proteins,^9,10^ vesicular stomatitis virus (VSV) based vectors^11–14^ or mRNA-delivered^15^ candidate vaccines have been shown in animal studies and have subsequently progressed to clinical trials. In particular, the sHeV-G recombinant protein vaccine represents a highly promising vaccine. It induced protective immunity across multiple animal models, notably in non-human primates, advancing to phase 1 clinical trials. However, challenges persist. The G proteins of NiV (NiV-G) and HeV (HeV-G) have ~80% amino acid homology, which to some extent limits their ability to induce cross-immune antibody responses.^23^ Therefore, overcoming the ability of natural G proteins to trigger enhanced cross-protection is crucial for the development of effective universal HNV vaccines.

The identification of neutralizing epitopes on the G and F proteins, as defined by their recognition by neutralizing antibodies, can contribute to the design of HNV antigens that induce broad cross-immunity.^28–38^ For example, the cross-immunity monoclonal antibody m102.4, which is isolated from a human phage display library, binds the cell receptor-binding domain of G proteins.^28,30,31^ It has been administered on an emergency compassionate basis to individuals with high-risk exposure to HeV or NiV infection and has gone through a phase 1 clinical trial in Australia.^32^ Other monoclonal antibodies, including HENV-26, HENV-32, and nAH1.3, which bind distinct epitopes on the cell receptor recognition site or the bottom side of the HNV G proteins, also demonstrated substantial protective efficacy.^29,34^ Several neutralizing monoclonal antibodies that target the prefusion conformation of the NiV/HeV fusion (F) glycoprotein and broadly neutralize henipaviruses ^35,36^have been identified. These mAbs bind conserved, glycan-sparse epitopes—especially at the membrane-distal apex and quaternary interfaces—blocking the conformational changes essential for fusion.^38,39^ Structural studies reveal the apex (domain III) as a vulnerable, functionally constrained site.^40^ Some mAbs target non-overlapping epitopes and exhibit synergy in cocktails to increase their breadth and limit escape.^41^ Several confer robust protection in animal models, supporting their therapeutic promise and guiding vaccine design.^37^

In this study, we identified a distinct vulnerable epitope on NiV-G specifically recognized by a potent neutralizing monoclonal antibody 14F8 and depicted crystal structure of the complex formed by this epitope and the 14F8 Fab fragment at atomic resolution. Leveraging structure-guided design, we introduced a single amino acid substitution (S586N) in HeV-G to reconstruct this NiV-specific epitope on HeV-G, which generated the HeV-G_S586N_ mutant. This new antigen was further characterized and tested for its cross-protection properties and immunogenicity against both NiV and HeV in mice and monkeys. Functional assays and immunogenicity studies in mice and non-human primates demonstrated that HeV-G_S586N_ elicited broad neutralizing responses against both HeV and NiV. Crystal structures further revealed that the S586N substitution induced a 9 Å conformational rearrangement in HeV-G, reshaping the protein framework and solvent-accessible surface area to mimic the NiV epitope. This work presents a structure-guided epitope reconstruction strategy that may help overcome cross-species antigenic differences within HNV and provides a potential approach for developing broad-spectrum immunogens.

## Results

### 14F8 recognizes a unique neutralizing epitope on the NiV-G protein

To identify novel neutralizing epitopes on NiV-G, we used hybridoma technology to isolate monoclonal antibodies from mice immunized with a soluble recombinant NiV-G protein (Bangladesh strain). One antibody, 14F8, was selected for further study because of its exceptional neutralizing activity and ability to define a NiV-specific vulnerable site. The variable regions (VH and VL) of m14F8 were cloned and assembled into a human IgG1 antibody constant region (CH and CL) backbone. The resulting chimeric antibody, 14F8, was expressed via the Expi293F expression system. SDS‒PAGE and size exclusion chromatography (SEC) confirmed that the chimeric 14F8 was highly pure (Supplementary Fig. 1).

**Fig. 1:**
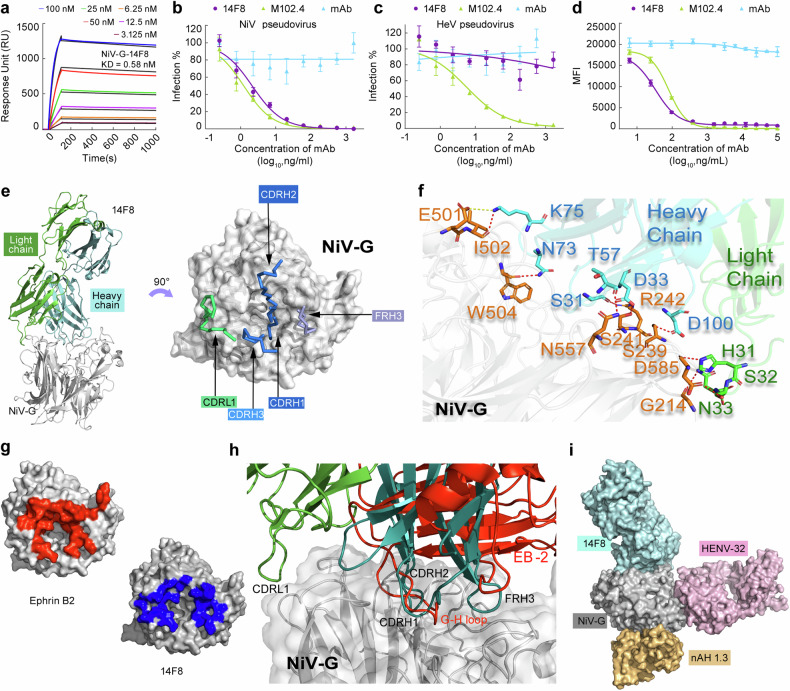
The monoclonal antibody 14F8 recognizes a unique vulnerable neutralization epitope on the NiV-G protein. **a** Kinetics of the binding of 14F8 to the NiV-G protein assessed by SPR on Biacore. The raw data are displayed in color, whereas the fitted data are shown in black. Comparison of the neutralization activities of 14F8, m102.4, and a nonspecific control antibody (anti-VSV-G, 8G5F11) against NiV pseudoviruses (**b**) and HeV pseudoviruses (**c**) in Ephrin B2-expressing 293T cells. Data represents percent neutralization against varying antibody concentrations; representative of three replicates. The data were processed via the inhibitor vs. response model, and the IC50 values were calculated. The fitted curves are displayed in the figure. **d** Neutralizing activity of 14F8, m102.4, and the control antibody against NiV as measured by a Luminex competitive inhibition assay. The data represent the mean fluorescence intensity (MFI) against varying antibody concentrations and are representative of two replicates. **e** Crystal structure of the 14F8 Fab–NiV-G complex. Residues from the 14F8 CDRH and CDRL and the framework regions (FRH) of the 14F8 heavy chain that are involved at the interaction interface are displayed as colored sticks. **f** Detailed interactions between 14F8 Fab and NiV-G. 14F8 heavy and light chains and belonging residues are shown in cyan and green, respectively, and NiV-G is shown in gray. The residues in NiV-G are in orange. The red dashed lines represent hydrogen bonds. **g** Contact surface of NiV-G with 14F8 (red) and the Ephrin B2 receptor (39) (blue), mapped on NiV-G (gray). **h** Alignment of the crystal structures of 14F8 Fab–NiV-G and the Ephrin B2-NiV-G complex (PDB ID: 2VSM). NiV-G, gray; 14F8 heavy and light chains, cyan and green, respectively; Ephrin B2, red. **i** Binding sites of 14F8, HENV-32, and nAH1.3

The affinity of 14F8 for HNV-G proteins was measured by SPR on a Biacore system. Humanized 14F8 retained a high affinity for NiV-G (*K*_D_ = 0.58 nM) (Fig. 1a), whereas it had no substantial binding capacity for HeV-G (Supplementary Fig. 1), and the removal of N-linked glycans from NiV-G had no measurable impact on the binding affinity between 14F8 and NiV-G (Supplementary Fig. 1). Next, we evaluated the neutralizing activity of 14F8 by performing a pseudovirus neutralization assay using HEK293 cells stably overexpressing Ephrin B2 and infected with HIV backbone-based pseudoviruses. In this system, the half maximal inhibitory concentration (IC50) of 14F8 against the NiV Bangladesh strain pseudovirus was 0.18 ng/mL (Fig. 1b), whereas 14F8 had no neutralizing activity against HeV pseudovirus (Fig. 1c). A Luminex receptor competition inhibition assay revealed an IC50 of 31.85 ng/mL against the NiV Bangladesh strain, which was close to that of m102.4 (76.24 ng/mL) (Fig. 1d). These results suggested that 14F8 is able to prevent NiV-G from attaching to its cellular receptor. Furthermore, the lack of binding and inhibition activity toward HeV-G revealed that 14F8 recognized a distinct vulnerable epitope on NiV-G.

To define the 14F8-binding epitope accurately, the crystal structure of the 14F8 Fab in complex with the NiV-G Bangladesh strain head domain (14F8 Fab–NiV-G) was solved at a resolution of 2.8 Å (Table 1). The interaction between NiV-G and 14F8 is shown in Fig. 1f and Supplementary Table 1. 14F8 bound the top of the G protein with a buried surface area of 1351.3 Å^2^ (Fig. 1e). The crystal structure revealed a unique site of NiV-G binding to 14F8. The binding region recognized by the 14F8 heavy chain partially overlaps with the Ephrin B2 binding site (Fig. 1g, h and Supplementary Fig. 3).

**Table 1:** Data collection and refinement statistics (molecular replacement)

	Nipah Virus attachment (G) glycoprotein in complex with neutralizing antibody 14F8	Hendra Virus attachment (G) glycoprotein mutant S586N	Hendra Virus attachment (G) glycoprotein mutant S586N in complex with neutralizing antibody 14F8
Data collection			
Space group	P 41 21 2	P 1 21 1	C 2 2 21
Cell dimensions			
* a*, *b*, *c* (Å)	125.96 125.96 317.26	110.84 257.01193.52	110.84 257.01193.52
α, β, γ (°)	90.00 90.00 90.00	90.00 97.69 90.00	90.00 90.00 90.00
Resolution (Å)	36.45–2.79	41.59–3.30	37.79–3.22
* R*_sym_ or *R*_merge_	0.10	0.148	0.214
* I*/σ*I*	1.75 (at 2.81 Å)	3.08 (at 3.32 Å)	1.53 (at 3.25 Å)
Completeness (%)	98.9	98.5	98.9
Redundancy	13.2 (10.4)	6.2 (5.7)	5.1 (4.6)
Refinement			
Resolution (Å)	36.23–2.80	41.59–3.3	37.06–3.22
No. reflections	63450	19266	44342
* R*_work_/*R*_free_	21.93/24.35	23.2/28.13	19.45/22.74
No. atoms	13483	6453	13414
Protein	13096	6354	13086
Ligand/ion	0	84	0
Water	117	236	328
* B*-factors	62.49	101.44	67.55
Protein	62.59	101.28	66.63
Ligand/ion		118.01	
R.m.s. deviations			
Bond lengths (Å)	0.004	0.003	0.004
Bond angles (°)	0.83	0.70	0.78
Ramachandran favored (%)	93.81	93.92	96.84
Ramachandran allowed (%)	6.19	6.08	3.16
Ramachandran outliers (%)	0	0	0

The data for each structure is collected and analyzed from a single crystal. Statistics for the highest-resolution shell are shown in parentheses

The interactions between the 14F8 heavy chain and NiV-G involve Asp100, Asp33, Ser31, Asn73, Lys75 and Thr57 in the 14F8 heavy chain, which bind Ser239, Ser241, Arg242, Trp504, Ile502 and Asn557, respectively, in NiV-G through hydrogen bonds (Supplementary Table 1). Interestingly, Asn73 and Lys75 in 14F8 FRH3 formed hydrogen bonds with NiV-G. The nitrogen atom (NZ) of Lys75 in the 14F8 heavy chain formed a salt bridge with the oxygen atom (OE2) of Glu501 in NiV-G (Fig. 1e, f).

Three amino acids in CDRL1 of 14F8 were involved in the interaction (Fig. 1e, f). The nitrogen atom (NE2) of His31 in 14F8 CDRL1 formed a hydrogen bond with the oxygen atom (O) of Asp585 in NiV-G. The oxygen atom (OG) of Ser32 in 14F8 CDRL1 formed a hydrogen bond with the oxygen atom (OD1) of Asp585 in NiV-G. The nitrogen atom (ND2) of Asn33 in 14F8 CDRL1 formed a hydrogen bond with the oxygen atom (O) of Gly214 in NiV-G.

The epitopes of 14F8 partially overlapped those of the neutralizing antibodies m102.4 and HENV-26 and were completely different from those of HENV-32 and nAH1.3 (Fig. 1i and Supplementary Fig. 3). 14F8’s light chain epitope is distinct from the binding sites of m102.4/HENV-26/HENV-32/nAH1.3 (Supplementary Fig. 4).

### Reconstruction of the 14F8-recognized epitope on HeV-G via structure-guided amino acid substitution

**Fig. 2:**
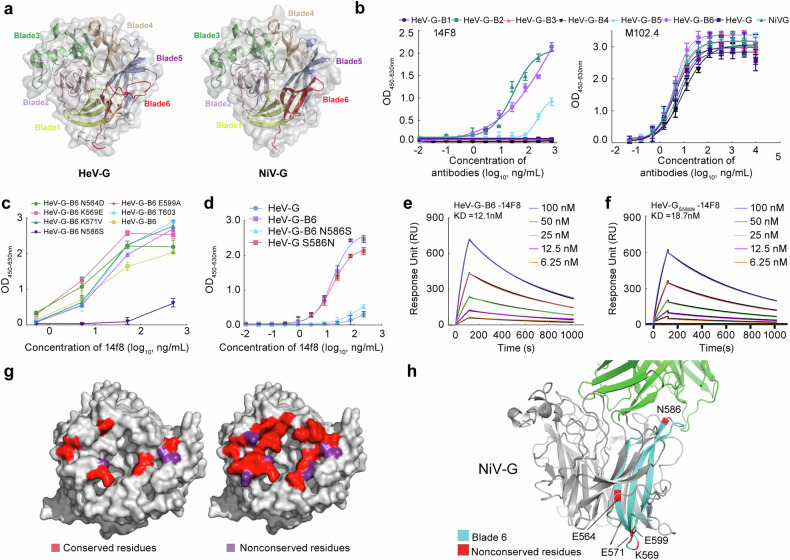
Reconstruction of the epitope of 14F8 on HeV-G via the S586N substitution. **a** The six-blade beta-propeller structure of the HeV-G and NiV-G proteins. **b**–**d** Affinity of 14F8 or m102.4 for various HeV-G mutants, as evaluated by ELISA. Data are representative of two technical replicates. Kinetics of the binding of 14F8 and HeV-G-B6 (**e**) or HeV-G_S586N_ (**f**) measured by Biacore. **g** Conservative analysis of the interactive surface (left) and the contact surface (right) between 14F8 and the NiV-G protein. Residues in the interactive surface are defined as those involved in direct interactions between two proteins. The contact surface refers to the solvent-accessible surface area of residues that become buried upon protein‒protein interaction. **h** Identification of conserved residues between the blade6 domains of the NiV-G protein. NiV-G, gray; blade6, cyan; nonconserved residues, red

HeV-G and NiV-G share a similar beta-propeller structure composed of six “blades” (Fig. 2a).^34,42^ As shown by the crystal structure, the recognized 14F8 is composed primarily of amino acid residues located on blades 1, 4, and 6 of the NiV-G β-propeller domains. To recreate this epitope on HeV-G, we constructed six mutants (HeV-G-B1 to B6) by replacing each blade of HeV-G with its corresponding blade in NiV-G (Supplementary Fig. 5). The recognition of each mutant by 14F8 was assessed via enzyme-linked immunosorbent assay (ELISA). Only HeV-G-B6 (including the mutation sites D564N, E569K, V571K, S586N, A599E, and S602T) was recognized by 14F8 (Fig. 2b), whereas all the mutants were recognized by the broadly neutralizing antibody m102.4, indicating that the HeV-G-B6 mutation did not alter the epitope recognized by m102.4 (Fig. 2b).

To further investigate the role of nonconserved amino acids in 14F8-G protein interactions, we constructed a series of single amino acid mutants based on HeV-G-B6. The six nonconserved amino acids between HeV-G and NiV-G on HeV-G-B6 were changed back one by one to wild-type HeV-G amino acids. Replacement of Asn586 with Ser in HeV-G-B6 generated the HeV-G-B6_N586S_ mutant, which was almost unable to bind 14F8. Moreover, all other mutants retained binding capacities to 14F8, similar to HeV-G-B6 (Fig. 2c). Because Ser586 is crucial for 14F8 recognition, a single amino acid mutation was performed in the HeV-G protein sequence to replace Ser586 with Asn. The resulting HeV-G_S586N_ mutant was expressed and purified, and its ability to bind 14F8 could be verified via ELISA (Fig. 2d). Moreover, 14F8 could bind HeV-G-B6 and HeV-G_S586N_ mutants with high affinity, with *K*_D_ values of 12.15 nM (Fig. 2e) and 18.72 nM (Fig. 2f), respectively, which were lower than those for either glycosylated or deglycosylated NiV-G.

Thus, the S586N mutation recapitulates the NiV-G epitope on the HeV-G scaffold by revealing the necessary contact residues. As shown by the crystal structure of the 14F8 Fab-NiV-G complex, the nonconserved amino acids in the B6 domain, except for N586, are located far from the recognition interface. Therefore, these factors were unlikely to influence 14F8 recognition. In contrast, N586 was located on the contact surface, and Asp585 adjacent to N586 interacted with His31 and Ser32 on 14F8 (Figs. 1f and 2g, h). The mutation of S586N significantly alters the binding interface in a way that permits 14F8 attachment. The ability of 14F8 to recognize and neutralize HeV pseudoviruses carrying the G protein S586N mutation further supports this conclusion (Supplementary Fig. 6). The yields of HeV-G and the HeV-G_S586N_ mutant were 12.7 mg/L and 15.5 mg/L, respectively, indicating that the S586N mutation did not adversely affect protein expression. SDS‒PAGE and SEC demonstrated the homogeneity and purity of HeV-G_S586N_ (Supplementary Fig. 7), HeV-G_S586N_ existed predominantly as a tetramer with a minor dimer population, whereas wild-type HeV-G comprised a mixture of tetramer, dimer, and monomer states. It has been previously reported that the natural oligomerization state of HeV-G does not significantly affect in vivo antigenicity.^43^

### HeV-G_S586N_ is broadly immunogenic against NiV and HeV

**Fig. 3:**
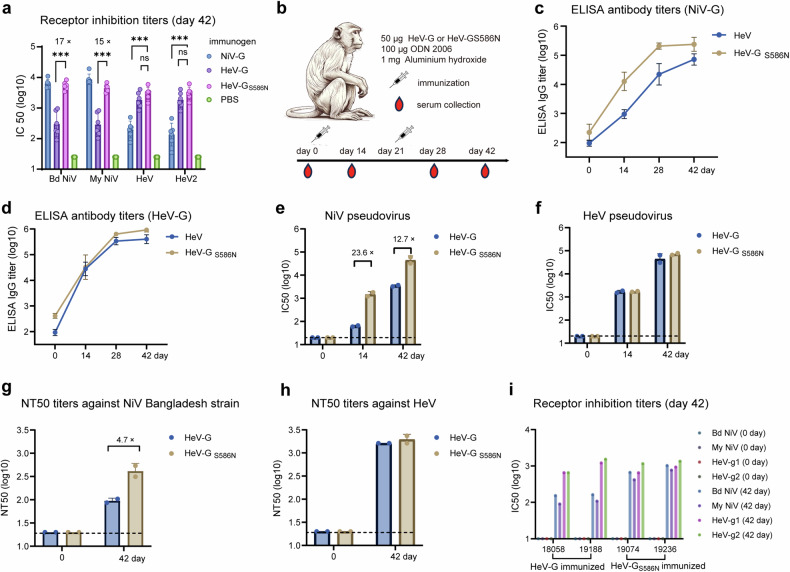
HeV-G_S586N_ elicits strong humoral immune responses against NiV and HeV in both mice and nonhuman primates. **a** Female BALB/c mice (6–8 weeks old, *n* = 6 per group) were immunized with 10 μg of NiV-G, HeV-G, or HeV-G_S586N_ protein supplemented with 200 μg of aluminum hydroxide and 20 μg of CpG 1826 on days 0 and 21. Mice immunized with PBS served as negative controls. On day 42 after the prime immunization, serum neutralizing antibodies were measured via a Luminex-based competitive inhibition assay against NiV-Bangladesh (Bd NiV), NiV-Malaysia (My NiV), HeV, and HeV2. **b** Male cynomolgus monkeys (4–5 years old, *n* = 2 per group) were immunized with 50 μg of HeV-G or HeV-G_S586N_ protein formulated with 1 mg of aluminum hydroxide and 100 μg of CpG 2006 on days 0 and 21. Serum binding antibody responses were assessed via ELISA (**c**, **d**, two technical replicates). Neutralizing activity was measured via pseudovirus neutralization assays (**e**, **f**, three technical replicates). Authentic virus neutralization assays were performed in a BSL-4 laboratory (**g**, **h**, four technical replicates). Receptor-binding inhibition was measured via Luminex-based competitive inhibition assays (**i**, two technical replicates). The IC50 values represent the dilution at which 50% infection or receptor binding is inhibited. NT50 values represent the reciprocal serum dilution at which 50% of virus infection is inhibited. The error bars indicate the standard deviation (SD) of biological replicates. Statistical significance was determined via unpaired two-tailed Student’s *t* tests, with *P < 0.05; **P < 0.01; and ***P < 0.001

To test whether HeV-G_S586N_ could trigger an enhanced cross-immune response, BALB/c mice were immunized with HeV-G_S586N_ with Alhydrogel and CpG 1826 as adjuvants. The receptor-inhibitory capacity of the elicited antibodies against NiV and HeV was tested via a Luminex multiplex assay. The receptor inhibition titer against NiV induced by HeV-G_S586N_ was significantly greater (~17-fold greater) than that induced by HeV-G. Meanwhile, the receptor inhibition titer against HeV elicited by HeV-G_S586N_ was similar to that induced by HeV-G (Fig. 3a). Thus, a single amino acid modification in the HeV-G sequence created an antigen capable of triggering a wider anti-HNV antibody response than a natural viral antigen.

Furthermore, the immunogenicity of HeV-G_S586N_ in nonhuman primates was evaluated (Fig. 3b–i). No preexisting anti-henipavirus neutralizing antibodies were detected prior to immunization. Fourteen days after the prime immunization, the titers of the Bangladesh strain NiV-G–specific antibody in the HeV-G_S586N_–immunized monkeys (IDs 19074 and 19236) reached 21,188 and 7558, respectively, whereas the titers in the HeV-G–immunized monkeys were 1212 and 743, respectively (Fig. 3c). HeV-G_S586N_ and HeV-G induced comparable levels of HeV (GenBank: AF017149.3) G-specific antibodies (Fig. 3d). Pseudovirus neutralization assays on day 14 demonstrated that the neutralizing titers in the HeV-G_S586N_ group were 23.6-fold greater than those in the HeV-G group (Fig. 3e). Following booster immunization, HeV-G_S586N_–immunized monkeys developed robust neutralizing antibody responses against NiV (Fig. 3e). By day 42, the IC50 titer in the HeV-G_S586N_ group was 12.7-fold greater than that in the HeV-G group. Live-virus neutralization assays further confirmed that, compared with wild-type HeV-G, the HeV-G_S586N_ group presented a 4.7-fold increase in neutralizing titers against NiV-Bangladesh (Fig. 3g). Moreover, the levels of HeV-neutralizing antibodies induced by HeV-G_S586N_ were comparable to those induced by wild-type HeV-G (Fig. 3f, h). Additionally, Luminex multiplex assays demonstrated that sera from HeV-G_S586N_–immunized monkeys exhibited strong and balanced receptor-blocking activity against NiV-Malaysia, NiV-Bangladesh, HeV, and HeV2 (Fig. 3i).

To further explore the antibody characteristics elicited by HeV-G_S586N_, we carried out a competitive inhibition experiment, and sera from HeV-G_S586N_-immunized monkeys presented stronger competition for 14F8 binding with NiV-G (EC50 = 58.4) (Supplementary Fig. 8a) than did those from wild-type HeV-G-immunized monkeys (EC50 = 7.7). Meanwhile the competition curves for HeV-G_S586N_-immunized sera against nAH1.3 and HENV32 showed limited shifts (2.7-fold and 1.8-fold, respectively) compared to HeV-G-immunized sera (Supplementary Fig. 8b-c). When bound to HeV-G, the competitive EC50 changes of nAH1.3 and HENV32 were 1.5-fold and 1.6-fold, respectively, 14F8 was not included because it did not bind to HeV-G (Supplementary Fig. 9). This finding indicates that HeV-G_S586N_ specifically induced 14F8-like antibodies without affecting the ability to elicit other neutralizing antibodies.

We also carried out a serum depletion assay in which excess HeV-G was used to deplete sera from immunized monkeys, followed by neutralization assays with the NiV pseudovirus. The results revealed a significant decrease in neutralizing activity in the HeV-G-immunized group (inhibition rate <50%), whereas sera from HeV-G_S586N_-immunized animals retained strong inhibitory activity (Supplementary Fig. 10). These findings suggest that antibodies induced by HeV-G moderately cross-recognize NiV-G, whereas HeV-G_S586N_ induces 14F8-like antibodies that specifically recognize NiV-G and are not depleted by HeV-G.

### S586N substitution induces epitope rearrangement on HeV-GS586N

**Fig. 4:**
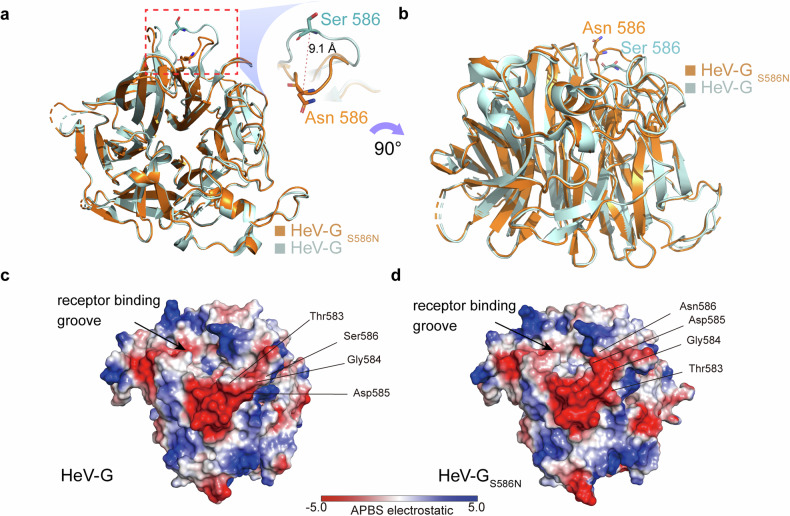
Comparison of the crystal structures of the wild-type and S586N-mutated HeV-G proteins. **a**, **b** Overlay of the crystal structures of wild-type (cyan) and mutant (orange) HeV-G. The red dotted line at higher magnification shows the measured distance between wild-type Ser 586 and substituted Asn 586 on the mutant. Comparison of the electrostatic surface potential on wild-type (**c**) and S586N-mutated HeV-G (**d**) proteins. The distinctive areas are indicated by black lines

To elucidate the molecular mechanism underlying the creation of a new epitope on HeV-G_S586N_, the crystal structure of the mutant protein was determined at 3.3 Å resolution (Table 1). and compared to that of wild-type HeV-G (RCSB Protein Data Bank ID: 2VSK). Except for blade6, all other domains were consistent, confirming the accuracy of the crystal structure. At position 586 of blade6, the nonconserved amino acid S586N was the closest to the 14F8 interacting surface. Compared with wild-type HeV-G, the single amino acid substitution caused the top of blade6 to shift toward the center of the receptor-binding surface by a maximum distance of 9 Å (Fig. 4a, b and Supplementary Fig. 11).

The substitution modified the local molecular framework to elicit relative shifts in the adjacent amino acids Asp585, Thr583, and Gly584 and changes in the solvent-accessible surface area (Fig. 4c, d). Therefore, even though only a single amino acid substitution was introduced, the protein conformation was significantly altered, which can explain the strikingly different antigenicity of the wild-type and mutant proteins. Although Asn586 was not directly involved in the interaction with the 14F8 antibody, shaping the molecular framework contributed to the formation of an immunodominant neutralizing epitope.

### Crystal structure of HeV-G_S586N_ in complex with 14F8

**Fig. 5:**
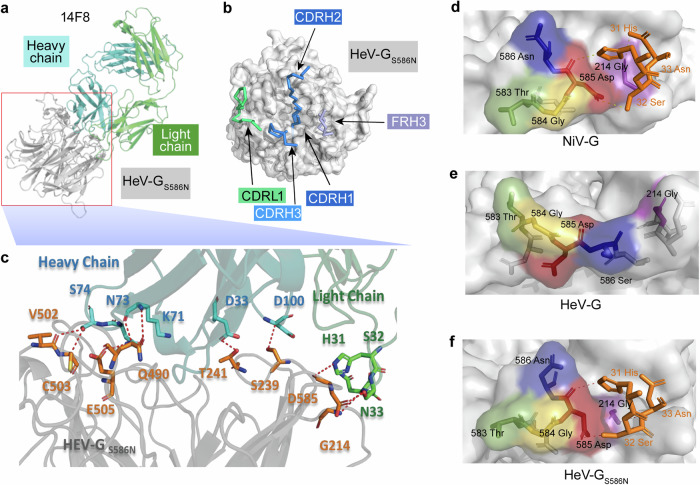
Crystal structure of the 14F8 Fab–HeV-G_S586N_ complex. **a**, **b** Crystal structure of the 14F8 Fab–HeV-G_S586N_ complex. Residues from CDRH1, CDRL, and FRH of 14F8 that are involved at the interaction interface are displayed as colored sticks. **c** Detailed interactions between 14F8 Fab and HeV-G_S586N_. The 14F8 heavy and light chains and belonging residues are shown in cyan and green, respectively, and HeV-G_S586N_ is in gray. The residues in HeV-G_S586N_ are in orange. The red dashed lines represent hydrogen bonds. 14F8-binding regions on NiV-G (**d**), HeV-G (**e**), and HeV-_GS586N_ (**f**); the amino acids forming the local binding grooves are displayed as sticks. Residues belonging to 14F8 CDRL1 are displayed in orange, and the hydrogen bonds are represented by orange dashes

To further prove that the vulnerable epitope recognized by 14F8 was successfully reconstructed, the crystal structure of the HeV-G_S586N_-14F8 Fab complex was solved at 3.2 Å resolution (Table 1, Fig. 5a, b and Supplementary Table 2). Although the overall structure of HeV-G_S586N_ was still significantly distinct from that of NiV-G, the S586N substitution triggered a conformational rearrangement at the top of blade6 that formed a binding groove very similar to that on NiV-G and reconstructed an epitope recognizable by 14F8.

The interaction between the 14F8 heavy chain and HeV-G_S586N_ involves Asp100, Asp33, Lys71 and Asn73 in 14F8, which bind Ser239, Thr241, Gln490 and Glu505, respectively, in HeV-G_S586N_ through hydrogen bonds. Ser74 in 14F8 forms hydrogen bonds with Val502 and Cys503 in HeV-G_S586N_ (Supplementary Table 2). These interactions contribute to the binding stability between 14F8 and HeV-G_S586N_ (Fig. 5c).

Three amino acids in the 14F8 light chain are involved in critical interactions for binding (Fig. 5c). The nitrogen atom (NE2) of His31 in 14F8 formed a hydrogen bond with the oxygen atom (O) of Asp585 in HeV-G_S586N_. The oxygen atom (OG) of Ser32 in 14F8 formed a hydrogen bond with the oxygen atom (OD1) of Asp585 in HeV-G_S586N_. The nitrogen atom (N) of Asn33 formed a hydrogen bond with the nitrogen atom (N) of Gly214 in HeV-G_S586N_.

In the HeV-G_S586N_-14F8 and NiV-G-14F8 complexes, His31, Ser32, and Asn33 in the CDRL1 of 14F8 created three hydrogen bonds with Asp 585 and Gly 214 in HeV-G_S586N_ and NiV-G (Fig. 5d). In contrast, in HeV-G, the presence of Ser586 results in a greater distance between the top of the blade6 domain and the receptor-binding surface, and the local conformation is markedly different from that of NiV-G (Fig. 5d–f). The oxygen atom of Asp585 could not be exposed at the appropriate position, which prevented the interaction between the 14F8 light chain and wild-type HeV-G.

Interestingly, some differential interactions were observed in the HeV-G_S586N_-14F8 and NiV-G-14F8 complexes, particularly in the heavy chain region. For example, the salt bridge between Lys75 of 14F8 FRH3 and Glu501 of NiV-G, the hydrogen bond between Ser31 of 14F8 CDRL1 and Arg242 of NiV-G, is absent in HeV-G_S586N_. Ser74 of 14F8 FRH3 formed hydrogen bonds with Cys503 and Val502 in HeV-G_S586N_, which are not observed in NiV-G. These variations highlight the subtle differences in how 14F8 interacts with NiV-G and HeV-G_S586N_.

## Discussion

NiV and HeV are two extremely dangerous and lethal pathogens, and no vaccine or treatment has yet been approved for humans. Vaccines are the most cost-effective method for controlling these fatal infections.^8^ The development of vaccines that are effective against both viruses would further reduce costs and streamline the approval process. Current vaccine candidates, particularly sHeV-G, have demonstrated promising immunogenicity, safety, and protective efficacy in multiple animal models.^10,44,45^ Enhancing NiV-neutralizing antibody responses through epitope reconstruction provides additional value by elucidating the mechanisms of NiV cross-neutralization induced by HeV-based immunogens while advancing antigen design methodologies. This approach may further improve the protective efficacy of sHeV-G or enable dose-sparing strategies for future public health needs. Several strategies have been explored to enhance cross-immunity against NiV and HeV, and an anti-CD40 monoclonal antibody fused with the NiV antigen induces cross-neutralizing responses against both NiV and HeV.^46^ The rational design of HeV-G_S586N_ in this study aims to enhance cross-neutralizing responses to NiV, supporting the development of a more effective universal HNV vaccine.

Here, we discovered a unique vulnerable epitope on the NiV attachment glycoprotein recognized by the potent neutralizing antibody 14F8. Several HNV monoclonal antibodies targeting G or F proteins have been reported.^28–38^ The 14F8 antibody recognizes a unique neutralizing epitope on NiV-G. Structural data show the 14F8 light chain binds a novel epitope not targeted by other antibodies. Crucially, this site differs between NiV and HeV at residue 586 (Asn in NiV, Ser in HeV). This single amino acid determines the divergent neutralization specificity observed for NiV and HeV. Such precise mapping of an antigenic determinant provides a clear model for studying G protein-directed neutralizing antibodies. It directly illustrates how minor structural variations dictate immune recognition, advancing our mechanistic understanding of immunogenicity.

This unique NiV-G neutralizing epitope was subsequently reconstructed on HeV-G to create the broad-spectrum antigen HeV-G_S586N_ via a structure-guided single amino acid substitution(S586N). This minimal modification could preserve the native neutralizing epitopes on HeV-G to the greatest extent while promoting high levels of anti-NiV and -HeV neutralizing antibodies. In preclinical testing, we demonstrated that HeV-G_S586N_ could enhance cross-immunity in both mice and monkeys, showing its potential as a universal antigen against HeV and NiV. Our study reveals an early and robust cross-neutralizing antibody response, with NiV-neutralizing antibodies detectable as early as 2 weeks postprime in monkeys immunized with HeV-G_S586N_. This rapid induction may reflect enhanced B-cell activation due to optimized epitope presentation by the mutant antigen. This study primarily focused on evaluating the humoral immune response induced by HeV-G_S586N_, which showed promising cross-neutralizing activity. However, the cellular immune response, including T cell activation and memory formation, was not systematically assessed in the current experiments. Further investigations are warranted to clarify the contribution of cellular immunity to the overall protective efficacy. While our data demonstrate strong initial cross-neutralization, the durability of this response remains to be fully assessed. Longitudinal studies tracking antibody titers over extended periods are currently underway. Future studies should also comprehensively evaluate the longevity of cross-reactive antibodies and protective immunity following HeV-G_S586N_ immunization, including exploring potential booster vaccination strategies, assessing immune memory responses upon re-exposure to viral antigens.

The molecular basis of the widened protection triggered by the S586N substitution in HeV-G was precisely characterized through the resolution of three crystal structures. Our findings provide a comprehensive understanding of how a single amino acid alteration can lead to significant changes in antigenicity and immunogenicity. Utilizing X-ray crystallography techniques, we successfully generated high-quality protein crystals and determined the crystal structure of the HeV-G_S586N_. We established an atomic-scale map of the key neutralizing epitopes on NiV-G and HeV-G_S586N_. Although the Ser-to-Asn at position 586 did not interact directly with 14F8, it influenced antibody binding and cross-immunity by altering the conformation of the protein. S586N substitution successfully induced conformational rearrangement and epitope reconstruction, which elicited more NiV-neutralizing antibodies against the epitope. Moreover, the conservation of HeV-G’s main sequence and structure stimulated a significant level of anti-HeV neutralizing antibodies. Taken together, these results demonstrate the viability of our rational design strategy. These results contribute to the understanding of structure-guided antigen design and may inform future efforts to develop vaccines for emerging and re-emerging viral pathogens.

The concept of this strategy involves the identification and utilization of neutralizing antibody-recognized epitopes across different viral strains. This process leverages structure-guided modifications to accurately reconstruct a neutralizing epitope from one viral strain onto another, thereby eliciting a broad-spectrum immune response. The principles of epitope reconstruction can be generalized to other pathogens, particularly those where cross-protection is highly desirable. For example, in the context of HIV,^47^ similar strategies can be explored when neutralizing antibodies against different strains have been identified and their corresponding epitopes have been mapped. By introducing specific mutations to reconstitute these epitopes on a core strain, researchers might systematically engineer immunodominant epitopes that elicit robust and cross-neutralizing antibody responses.

In this study, we evaluated the immunogenicity of HeV-G_S586N_ in mice and Cynomolgus monkeys. While the results demonstrated its potential to induce robust neutralizing antibody responses, there are several limitations. Our NHP immunogenicity findings are preliminary because of the limited group size, and a larger cohort of animals will be necessary to rigorously assess protective efficacy of HeV-G_S586N_ and to allow for statistical comparisons. Although cynomolgus monkeys are commonly used and share immunological similarities with humans, owing to the absence of a lethal model, in vivo challenge experiments will be conducted in African green monkeys.

In conclusion, our study provides an effective, broad-spectrum antigen for NiV and HeV prevention and concurrently provides a proof-of-concept for the use of epitope reconstruction in universal vaccine design. The success of HeV-G_S586N_ demonstrates that rational design strategies based on structural biology can yield antigens with enhanced cross-immunogenicity, offering a promising alternative to traditional vaccine development approaches. Future research could build on these findings by exploring the optimization of epitope reconstruction techniques, evaluating the long-term durability of immune responses elicited by similar antigens, and investigating the feasibility of applying this concept to other virus families with high pandemic potential.

## Materials and methods

### Protein expression and purification

The extracellular and head domains of HeV (APT69530), HeV2 (QYC64605), NiV Bangladesh isolate (AAY43916), and NiV Malaysia isolate (AAK29088) G proteins were optimized for codon usage. A tPA signal peptide and a Strep-II tag were fused at the N-terminal region. These optimized sequences were synthesized (General Biosystems Co., Ltd, China) and subsequently cloned into the pcDNA3.1 (+) vector. Expi-293F cells were transfected with the constructed plasmids and maintained under shaking conditions (120 rpm, 37 °C). Five days post-transfection, supernatants were harvested by centrifugation at 3000 × *g* for 15 min and further clarified using a 0.45 μm sterile filter (Thermo Fisher Scientific, USA). Proteins were isolated by affinity chromatography using StrepTrap HP columns (GE Healthcare, USA), then concentrated via 30K ultrafiltration tubes (Merck Millipore, Germany), and exchanged into phosphate-buffered saline (PBS, pH 7.4). For the preparation of deglycosylated proteins, purified recombinant G proteins were enzymatically deglycosylated with PNGase F at 37 °C for 12 h. The quality and concentration of final purified proteins were verified through SDS-PAGE and BCA Protein Assay Kit (Thermo Fisher Scientific, USA), respectively.

### Monoclonal antibody generation, isolation, and chimeric antibody production

Purified recombinant NiV-G protein was emulsified with Freund’s complete adjuvant at equal volume and administered subcutaneously into multiple sites in the mice groin area, each receiving a total of 20 μg protein. Booster immunizations were carried out at 2-week intervals, employing Freund’s incomplete adjuvant. Serum antibody titers were quantified by ELISA 2 weeks after the third immunization. Splenocytes harvested from immunized mice were fused to Sp2/0 myeloma cells at a ratio of 1:10 and cultured in aminopterin-thymidine selection medium for 10 days, then switched to HT medium. Initial positive clones were isolated and repeatedly subcloned to confirm their specificity. Hybridomas were injected intraperitoneally into mice to generate ascites fluids. Antibody titers from ascites and hybridoma supernatants were determined by ELISA. The antibody variable regions were sequenced by 5’ RACE, analyzed using IMGT/V-QUEST, and cloned into human IgG1 backbone in the pcDNA3.1 vector. Chimeric antibody 14F8 and reported antibody m102.4^48^ were expressed in Expi293F cells and purified using Protein A affinity chromatography (GE Healthcare). Purified antibodies were characterized by SDS-PAGE and SEC.

### Surface plasmon resonance

The kinetics of antibody‒antigen binding were measured via surface plasmon resonance (SPR) on a Biacore T200 (GE Health Care, USA). Before the experiment, the antibody was diluted to 0.5 μg/mL in HBS-EP + buffer (GE Health Care, USA). During the experiment, the protein A chip was run at a flow rate of 10 μL/min for 60 s, whereas the G proteins were tested in serial dilutions from 100 nM at a flow rate of 30 μL/min. The flow durations for association and dissociation were 120 s and 900 s, respectively.

### Animal immunization

All animal studies were approved by the Animal Welfare and Ethics Committee of Academy of Military Medical Sciences (Permission Nos.: IACUC-SWGCYJS-2020-03, IACUC-DWZX-2023-043). Female BALB/c mice (6–8 weeks, SPF grade) from Beijing Viton Lever Laboratory Animal Technology Co. were immunized intramuscularly in hind limbs with 10 μg recombinant protein plus 200 μg aluminum and 20 μg CpG 1826 in 100 μL; control mice received PBS. Tail vein blood was collected 42 days post-immunization for serum analysis.

Four male cynomolgus monkeys (4–5 years old) from Beijing Institute of Biotechnology’s animal center were divided into two groups. Each received 50 μg protein adjuvanted with 1 mg aluminum and 100 μg CpG 2006 in 500 μL via hind limb i.m. injection on days 0 and 21. Blood sampling occurred on vaccination days 0, 14, 28, and 42.

### Enzyme-linked immunosorbent assay (ELISA)

Purified G proteins (1 μg/mL in 50 mM carbonate buffer, pH 9.6) were coated onto 96-well plates by overnight incubation at 4 °C. Plates were washed thrice with PBS containing 0.1% Tween-20 (PBST), then blocked with PBS containing 2% BSA (100 μL/well) for 1 h at 37 °C. After blocking buffer removal, serially diluted sera in PBS with 0.2% BSA were added in duplicate wells and incubated 1 h at 37 °C. Following PBST washes, horseradish peroxidase (HRP)-conjugated goat anti-mouse IgG (Abcam, UK; 1:50,000) or goat anti-monkey IgG HRP (Abcam, UK; 1:20,000) was added (100 μL/well) and incubated 45 min at 37 °C to detect species-specific antibodies. Post-incubation, plates were washed, and 100 μL/well TMB substrate (Solarbio Life Sciences, China) was added. After 5 min incubation, reactions were stopped with 50 μL/well Stop Solution (Solarbio Life Sciences, China). Absorbance was measured at 450 nm with 630 nm reference.

### Serum competition ELISA

Plates were coated with purified G proteins (2 μg/mL in 50 mM carbonate buffer, pH 9.6) overnight at 4 °C. After PBST washing, blocking was performed using 2% BSA in PBS (100 μL/well, 1 h, 37 °C). Duplicate wells received 1:3 serially diluted monkey sera incubated 30 min at 37 °C. Primary amine-biotinylated IgG antibodies (14F8, nAH1.3, HENV-32) were then added separately, followed by 30 min incubation at 37 °C. Post-wash, HRP-conjugated streptavidin (Abcam, UK; 1:4000, 100 μL/well) was added and incubated 30 min at room temperature. After final washing, TMB substrate (100 μL/well; Solarbio Life Sciences, China) was incubated for 5 min before reaction termination with Stop Solution (50 μL/well; Solarbio Life Sciences, China). Absorbance (450 nm/630 nm) was measured.

### Luminex receptor competition inhibition assay

Luminex magnetic beads coated with the HNV G proteins G_NiV-Bd_, G_NiV-My_, and G_HeV_ were packed as previously described.^23,49^ Serum samples from vaccinated animals were serially diluted in PBS containing 1% BSA. Aliquots (20 μL) of each dilution were added in duplicate to black opaque 96-well plates, followed by addition of 1500 antigen-coupled beads per well. Plates were incubated at 800 rpm for 1 h at room temperature. Subsequently, 10 μL of biotinylated mouse Ephrin B2 Fc chimera (R&D Systems; final concentration: 16.5 ng/mL) was added to each well with incubation at 800 rpm for 30 min. After adding 10 μL of streptavidin-R-phycoerythrin (SAPE, 12 μg/mL), plates were placed on a magnetic separator (Luminex) for bead immobilization. Following three washes with 1% BSA-PBS, bead-associated fluorescence was quantified using a Luminex MAGPIX system. Assay validation included EFNB2 binding and m102.4 inhibition controls (Supplementary Fig. 2).

### Pseudovirus neutralization assay

The HIV-based pseudoviruses were generated following the method described previously.^23^ The codon-optimized NiV G and F genes (General Biologics Ltd, China) were inserted into pcDNA3.1(+) to obtain envelope-expressing plasmids (pcDNA3.1-G and pcDNA3.1-F) for the HIV pseudovirus backbone. Similarly, the codon-optimized HeV-G and HeV-F genes were cloned into the same vectors for HeV pseudovirus preparation. Briefly, 4.0 × 10^6^ 293T cells were plated in 10-cm culture dishes 24 h before transfection. Transfection involved co-introduction of envelope plasmids (pcDNA3.1-G and pcDNA3.1-F), the HIV backbone plasmid pNL4-3.Luc-R-E, and Lipofectamine 3000 (Invitrogen). To prepare the GS586N pseudovirus variant, wild-type HeV-G was replaced by the S586N-mutated plasmid during transfection. At 60 h post-transfection, culture supernatants were collected, centrifuged at 3500 × *g* for 15 min, filtered through a 0.45 μm membrane, and stored at −80 °C.

Animal sera from vaccinated subjects were heat-treated at 56 °C for 30 min, serially diluted in 96-well plates, and incubated with 50 μL pseudovirus per well for 1 h at 37 °C. Subsequently, 293T cells (6 × 10^4^ cells/well, in 50 μL) were added, incubated for an additional 48 h at 37 °C, and luciferase signals were measured with a GloMax System (Promega, USA). IC50 values were determined using the inhibitor-response curve fitting model in Prism 9.0 software.

### Neutralization assay

Neutralization assays were performed in BSL-4 containment using Vero E6 cells in 96-well plates. Sera underwent threefold serial dilution in DMEM with 2.5% FBS and were mixed 1:1 with virus (100 TCID_50_/well). After 1 h incubation at 37 °C, 100 μL mixtures were transferred to confluent Vero E6 monolayers for 1 h. The inoculum was removed; then 200 μL DMEM containing 2.5% FBS and 0.9% methylcellulose was added per well. Plates were incubated at 37 °C/5% CO_2_ for 5 days. Quadruplicate wells were scored as CPE-positive (+) or negative (−), with 50% neutralizing titer (NT50) calculated by the Reed-Muench method against NiV (Bangladesh strain, CSTR:16533.06. IVCAS 6.7488) and HeV (CSTR:16533.06. IVCAS 6.7487).

### Neutralization assay with depleted monkey sera

Monkey serum was added in duplicate to 96-well plates, followed by HeV-G (2 μg/well) in a 100 μL total volume for 37 °C incubation (30 min). Subsequently, 50 μL pseudovirus was added per well with further incubation (37 °C, 1 h), after which 50 μL of 293T cell suspension (6 × 10^4^ cells/well) was introduced. Plates were cultured for 48 h (37 °C), maintaining a final serum concentration of 1:600. Luciferase signals were quantified using the Glomax Luciferase Assay System (Promega, USA).

### Crystallization and data collection

For crystallization, the extracellular head domain of NiV-G (NP_112027) was increased with a tPA signal peptide at its N-terminus and a 6× His tag at its C-terminus. The plasmid was transfected into Expi-293F cells for expression. The protein was purified via Ni affinity chromatography. The recombinant protein was incubated with PNGase for 12 h at 37 °C for deglycosylation. The extracellular head domain of the HeV-G_S586N_ protein was increased with a tPA signal peptide and a StrepII tag to its N-terminus. The recombinant protein was expressed in Expi-293F cells and purified on a StrepTrap HP affinity column (GE Health Care, USA).

For 14F8 Fab crystallization, the light and heavy chains of 14F8 were used. A tPA signal peptide was added to the N-terminus, and a 6× His tag was added to the C-terminus of the heavy chain. The protein was expressed in Expi-293F cells and purified via Ni affinity chromatography. The 14F8 Fab-NiV-G complex and 14F8 Fab-HeV-G_S586N_ complex were obtained by coincubating the Fab and G proteins at 37 °C for 4 h, followed by purification by molecular exclusion chromatography.

14F8 Fab–NiV-G complex crystals were successfully grown at 16 °C in sitting drops over wells containing 2.05 M ammonium sulfate, 0.1 M BIS-Tris, and 10 mM barium chloride dihydrate, pH 5.5. The drops were produced by mixing 200 nL of the 14F8 Fab-NiV-G complex (18.3 mg/mL) in 20 mM HEPES (pH 7.0) or 150 mM NaCl with 200 nL of well solution. The crystals were flash cooled in liquid nitrogen with 4 M sodium malonate for cryoprotection.

The HeV-G_S586N_ crystals were successfully grown at 16 °C in sitting drops over wells containing 12.5–13.5% PEG 3350 and 100 mM NaAc, pH 4.7. The drops were produced by mixing 200 nL of HeV-G_S586N_ (16 mg/mL) in 20 mM HEPES, pH 7.0, 150 mM NaCl with 200 nL of well solution. The crystals were flash-cooled in liquid nitrogen with 20% glycerin cryoprotection.

14F8 Fab–HeV–G_S586N_ complex crystals were successfully grown at 16 °C in sitting drops over wells containing 13–14% PEG 3350, 10 mM MgCl_2_, and 5 mM NiCl_2_, pH 6.5 PIPES. The drops were produced by mixing 200 nL of the 14F8 Fab-HeV-G_S586N_ complex (15.3 mg/mL) in 20 mM HEPES (pH 7.0) or 150 mM NaCl with 200 nL of well solution. The crystals were flash-cooled in liquid nitrogen with 20% glycerin cryoprotection.

Diffraction data were collected on the BL02U1 beam line of the Shanghai Synchrotron Radiation Facility at a wavelength of 1.07180 Å. The diffraction temperature was 100 K. The diffraction data were automatically processed via the Aquarium pipeline24.

The interface residues were analyzed via the MOE v2014.1001 protein contact module. The molecular graphics were generated by PyMOL.

### Statistical analysis

All the statistical analyses were performed with GraphPad Prism 9.0.0 software. The antibody titers were transformed to log10 values before analysis. The unpaired two-tailed Student’s *t* test was used to analyze differences between groups; *P* < 0.05 was considered significant (*P < 0.05; **P < 0.01; ***P < 0.001).

## Supplementary information

Supplemental material

## Data Availability

The atomic coordinates and structure factors have been deposited in the Protein Data Bank (PDB) under accession codes 8JA5, 8JR3, and 8JR5. All other data supporting the findings of this study are included in this published article and its supplementary information files.

## References

[1] Gurley, E. S., Spiropoulou, C. F. & de Wit, E. Twenty years of nipah virus research: where do we go from here? *J. Infect. Dis.* **221**, S359–S362 (2020). 32392321 10.1093/infdis/jiaa078 PMC7213572

[2] Murray, K. et al. A morbillivirus that caused fatal disease in horses and humans. *Science* **268**, 94–97 (1995). 7701348 10.1126/science.7701348

[3] Peel, A. J. et al. Novel Hendra virus variant circulating in black flying foxes and grey-headed flying foxes, Australia. *Emerg. Infect. Dis.* **28**, 1043–1047 (2022). 35447052 10.3201/eid2805.212338 PMC9045453

[4] Annand, E. J. et al. Novel Hendra virus variant detected by sentinel surveillance of horses in Australia. *Emerg. Infect. Dis.* **28**, 693–704 (2022). 35202527 10.3201/eid2803.211245 PMC8888208

[5] Chua, K. B. et al. Fatal encephalitis due to Nipah virus among pig-farmers in Malaysia. *Lancet* **354**, 1257–1259 (1999). 10520635 10.1016/S0140-6736(99)04299-3

[6] Centers for Disease Control and Prevention (C. D. C.) Outbreak of Hendra-like virus--Malaysia and Singapore, 1998-1999. *Morb. Mortal. Wkly. Rep.* **48**, 265–269 (1999). 10227800

[7] Mire, C. E. et al. Pathogenic differences between Nipah virus Bangladesh and Malaysia strains in primates: Implications for antibody therapy. *Sci. Rep.* **6**, 30916 (2016). 27484128 10.1038/srep30916 PMC4971471

[8] Gómez Román, R. et al. Medical countermeasures against henipaviruses: a review and public health perspective. *Lancet Infect. Dis.* **22**, e13–e27 (2022). 34735799 10.1016/S1473-3099(21)00400-X PMC8694750

[9] Mire, C. E. et al. A recombinant Hendra virus G glycoprotein subunit vaccine protects nonhuman primates against Hendra virus challenge. *J. Virol.* **88**, 4624–4631 (2014). 24522928 10.1128/JVI.00005-14 PMC3993805

[10] Bossart, K. N. et al. A Hendra virus G glycoprotein subunit vaccine protects African green monkeys from Nipah virus challenge. *Sci. Transl. Med.* **4**, 146ra107 (2012). 22875827 10.1126/scitranslmed.3004241 PMC3516289

[11] Foster, S. L. et al. A recombinant VSV-vectored vaccine rapidly protects nonhuman primates against lethal Nipah virus disease. *Proc. Natl. Acad. Sci. USA* **119**, e2200065119 (2022). 35286211 10.1073/pnas.2200065119 PMC8944267

[12] de Wit, E. et al. Distinct VSV-based Nipah virus vaccines expressing either glycoprotein G or fusion protein F provide homologous and heterologous protection in a nonhuman primate model. *EBioMedicine* **87**, 104405 (2023). 36508878 10.1016/j.ebiom.2022.104405 PMC9763366

[13] Mire, C. E. et al. Use of single-injection recombinant vesicular stomatitis virus vaccine to protect nonhuman primates against lethal Nipah virus disease. *Emerg. Infect. Dis.* **25**, 1144–1152 (2019). 31107231 10.3201/eid2506.181620 PMC6537706

[14] Lo, M. K. et al. Single-dose replication-defective VSV-based Nipah virus vaccines provide protection from lethal challenge in Syrian hamsters. *Antivir. Res.* **101**, 26–29 (2014). 24184127 10.1016/j.antiviral.2013.10.012 PMC3874889

[15] Loomis, R. J. et al. Chimeric fusion (F) and attachment (G) glycoprotein antigen delivery by mRNA as a candidate Nipah vaccine. *Front. Immunol.* **12**, 772864 (2021). 34956199 10.3389/fimmu.2021.772864 PMC8692728

[16] Defang, G. N., Khetawat, D., Broder, C. C. & Quinnan, G. V. Jr Induction of neutralizing antibodies to Hendra and Nipah glycoproteins using a Venezuelan equine encephalitis virus in vivo expression system. *Vaccine* **29**, 212–220 (2010). 21050901 10.1016/j.vaccine.2010.10.053 PMC3032421

[17] Kong, D. et al. Newcastle disease virus-vectored Nipah encephalitis vaccines induce B and T cell responses in mice and long-lasting neutralizing antibodies in pigs. *Virology* **432**, 327–335 (2012). 22726244 10.1016/j.virol.2012.06.001

[18] Ploquin, A. et al. Protection against henipavirus infection by use of recombinant adeno-associated virus-vector vaccines. *J. Infect. Dis.* **207**, 469–478 (2013). 23175762 10.1093/infdis/jis699 PMC7107322

[19] Guillaume-Vasselin, V. et al. Protection from Hendra virus infection with Canarypox recombinant vaccine. *npj Vaccines* **1**, 16003 (2016). 29263849 10.1038/npjvaccines.2016.3 PMC5707888

[20] Keshwara, R. et al. Rabies-based vaccine induces potent immune responses against Nipah virus. *npj Vaccines* **4**, 15 (2019). 31016033 10.1038/s41541-019-0109-5 PMC6465360

[21] van Doremalen, N. et al. A single-dose ChAdOx1-vectored vaccine provides complete protection against Nipah Bangladesh and Malaysia in Syrian golden hamsters. *PLoS Negl. Trop. Dis.* **13**, e0007462 (2019). 31170144 10.1371/journal.pntd.0007462 PMC6581282

[22] Loomis, R. J. et al. Structure-based design of Nipah virus vaccines: a generalizable approach to paramyxovirus immunogen development. *Front. Immunol.* **11**, 842 (2020). 32595632 10.3389/fimmu.2020.00842 PMC7300195

[23] Li, Y. et al. Fc-based recombinant henipavirus vaccines elicit broad neutralizing antibody responses in mice. *Viruses* **12**, 480 (2020). 32340278 10.3390/v12040480 PMC7232446

[24] Walpita, P. et al. Vaccine potential of Nipah virus-like particles. *PLoS ONE* **6**, e18437 (2011). 21494680 10.1371/journal.pone.0018437 PMC3071823

[25] Walpita, P. et al. A VLP-based vaccine provides complete protection against Nipah virus challenge following multiple-dose or single-dose vaccination schedules in a hamster model. *npj Vaccines* **2**, 21 (2017). 29263876 10.1038/s41541-017-0023-7 PMC5627259

[26] Stroh, E. et al. Henipavirus-like particles induce a CD8 T cell response in C57BL/6 mice. *Vet. Microbiol.* **237**, 108405 (2019). 31561922 10.1016/j.vetmic.2019.108405

[27] Middleton, D. et al. Hendra virus vaccine, a one health approach to protecting horse, human, and environmental health. *Emerg. Infect. Dis.* **20**, 372–379 (2014). 24572697 10.3201/eid2003.131159 PMC3944873

[28] Xu, K. et al. Crystal structure of the Hendra virus attachment G glycoprotein bound to a potent cross-reactive neutralizing human monoclonal antibody. *PLoS Pathog.* **9**, e1003684 (2013). 24130486 10.1371/journal.ppat.1003684 PMC3795035

[29] Dong, J. et al. Potent henipavirus neutralization by antibodies recognizing diverse sites on Hendra and Nipah virus receptor binding protein. *Cell* **183**, 1536–1550.e1517 (2020). 33306954 10.1016/j.cell.2020.11.023 PMC7771633

[30] Geisbert, T. W. et al. Therapeutic treatment of Nipah virus infection in nonhuman primates with a neutralizing human monoclonal antibody. *Sci. Transl. Med.* **6**, 242ra282 (2014). 10.1126/scitranslmed.3008929 PMC4467163 24964990

[31] Bossart, K. N. et al. A neutralizing human monoclonal antibody protects against lethal disease in a new ferret model of acute nipah virus infection. *PLoS Pathog.* **5**, e1000642 (2009). 19888339 10.1371/journal.ppat.1000642 PMC2765826

[32] Playford, E. G. et al. Safety, tolerability, pharmacokinetics, and immunogenicity of a human monoclonal antibody targeting the G glycoprotein of henipaviruses in healthy adults: A first-in-human, randomised, controlled, phase 1 study. *Lancet Infect. Dis.* **20**, 445–454 (2020). 32027842 10.1016/S1473-3099(19)30634-6

[33] Byrne, P. O. et al. Structural basis for antibody recognition of vulnerable epitopes on Nipah virus F protein. *Nat. Commun.* **14**, 1494 (2023). 36932063 10.1038/s41467-023-36995-y PMC10021056

[34] Wang, Z. et al. Architecture and antigenicity of the Nipah virus attachment glycoprotein. *Science* **375**, 1373–1378 (2022). 35239409 10.1126/science.abm5561 PMC12303162

[35] Dang, H. V. et al. An antibody against the F glycoprotein inhibits Nipah and Hendra virus infections. *Nat. Struct. Mol. Biol.* **26**, 980–987 (2019). 31570878 10.1038/s41594-019-0308-9 PMC6858553

[36] Dang, H. V. et al. Broadly neutralizing antibody cocktails targeting Nipah virus and Hendra virus fusion glycoproteins. *Nat. Struct. Mol. Biol.* **28**, 426–434 (2021). 33927387 10.1038/s41594-021-00584-8 PMC12334189

[37] Mire, C. E. et al. A cross-reactive humanized monoclonal antibody targeting fusion glycoprotein function protects ferrets against lethal Nipah virus and Hendra virus infection. *J. Infect. Dis.* **221**, S471–S479 (2020). 31686101 10.1093/infdis/jiz515 PMC7199785

[38] Avanzato, V. A. et al. A structural basis for antibody-mediated neutralization of Nipah virus reveals a site of vulnerability at the fusion glycoprotein apex. *Proc. Natl. Acad. Sci. USA* **116**, 25057–25067 (2019). 31767754 10.1073/pnas.1912503116 PMC6911215

[39] Byrne, P. O. et al. Structural and functional characterization of potent cross-neutralizing antibodies targeting henipavirus fusion glycoprotein. *Cell Host Microbe* **31**, 210–225 (2023).

[40] Wiley, D. C. et al. Prefusion-stabilized F glycoprotein of henipavirus reveals conserved neutralizing epitopes. *Sci. Transl. Med.* **16**, eabf1456 (2024).

[41] Xu, K. et al. Cooperative antibody binding to Henipavirus F reveals mechanisms of synergistic neutralization. *Immunity* **55**, 1054–1068.e1056 (2022).

[42] Bowden, T. A. et al. Structural basis of Nipah and Hendra virus attachment to their cell-surface receptor ephrin-B2. *Nat. Struct. Mol. Biol.* **15**, 567–572 (2008). 18488039 10.1038/nsmb.1435

[43] Bossart, K. N. et al. Receptor binding, fusion inhibition, and induction of cross-reactive neutralizing antibodies by a soluble G glycoprotein of Hendra virus. *J. Virol.* **79**, 6690–6702 (2005). 15890907 10.1128/JVI.79.11.6690-6702.2005 PMC1112112

[44] Geisbert, T. W. et al. A single dose investigational subunit vaccine for human use against Nipah virus and Hendra virus. *npj Vaccines* **6**, 23 (2021). 33558494 10.1038/s41541-021-00284-w PMC7870971

[45] Pallister, J. A. et al. Vaccination of ferrets with a recombinant G glycoprotein subunit vaccine provides protection against Nipah virus disease for over 12 months. *Virol. J.* **10**, 237 (2013). 23867060 10.1186/1743-422X-10-237 PMC3718761

[46] Pastor, Y. et al. A vaccine targeting antigen-presenting cells through CD40 induces protective immunity against Nipah disease. *Cell Rep. Med.* **5**, 101467 (2024). 38471503 10.1016/j.xcrm.2024.101467 PMC10983108

[47] Xu, K. et al. Epitope-based vaccine design yields fusion peptide-directed antibodies that neutralize diverse strains of HIV-1. *Nat. Med.* **24**, 857–867 (2018). 29867235 10.1038/s41591-018-0042-6 PMC6358635

[48] Zhu, Z. et al. Potent neutralization of Hendra and Nipah viruses by human monoclonal antibodies. *J. Virol.* **80**, 891–899 (2006). 16378991 10.1128/JVI.80.2.891-899.2006 PMC1346873

[49] Bossart, K. N. et al. Neutralization assays for differential henipavirus serology using Bio-Plex Protein Array Systems. *J. Virol. Methods* **142**, 29–40 (2007). 17292974 10.1016/j.jviromet.2007.01.003

