# Supplementary material for: Single amino acid substitution in Hendra virus attachment glycoprotein induces cross-neutralizing antibodies against Nipah virus
Source: Signal Transduct Target Ther. 2025 Aug 29;10:276. doi: 10.1038/s41392-025-02370-0 (PMC12394453; doi:10.1038/s41392-025-02370-0)
Supplement: Supplementary file 1 — Supplemental material [file 41392_2025_2370_MOESM1_ESM.docx]

Supplementary Materials for

Single amino acid substitution in Hendra virus attachment glycoprotein induces cross-neutralizing antibodies against Nipah virus

Yaohui Li#, Xiaoyan Huang#, Ruihua Li#, Xiaodong Zai#, Yilong Yang, Yue Zhang, Zhang Zhang, Jun Zhang, Junjie Xu*, and Wei Chen*

# These authors contributed equally to this work.

Correspondence to: xujunjie@sina.com (J.X.); cw0226@foxmail.com (W.C.)

**This PDF file includes:**

Figures. S1 to S11

Tables S1 to S2

**Figure S1.**
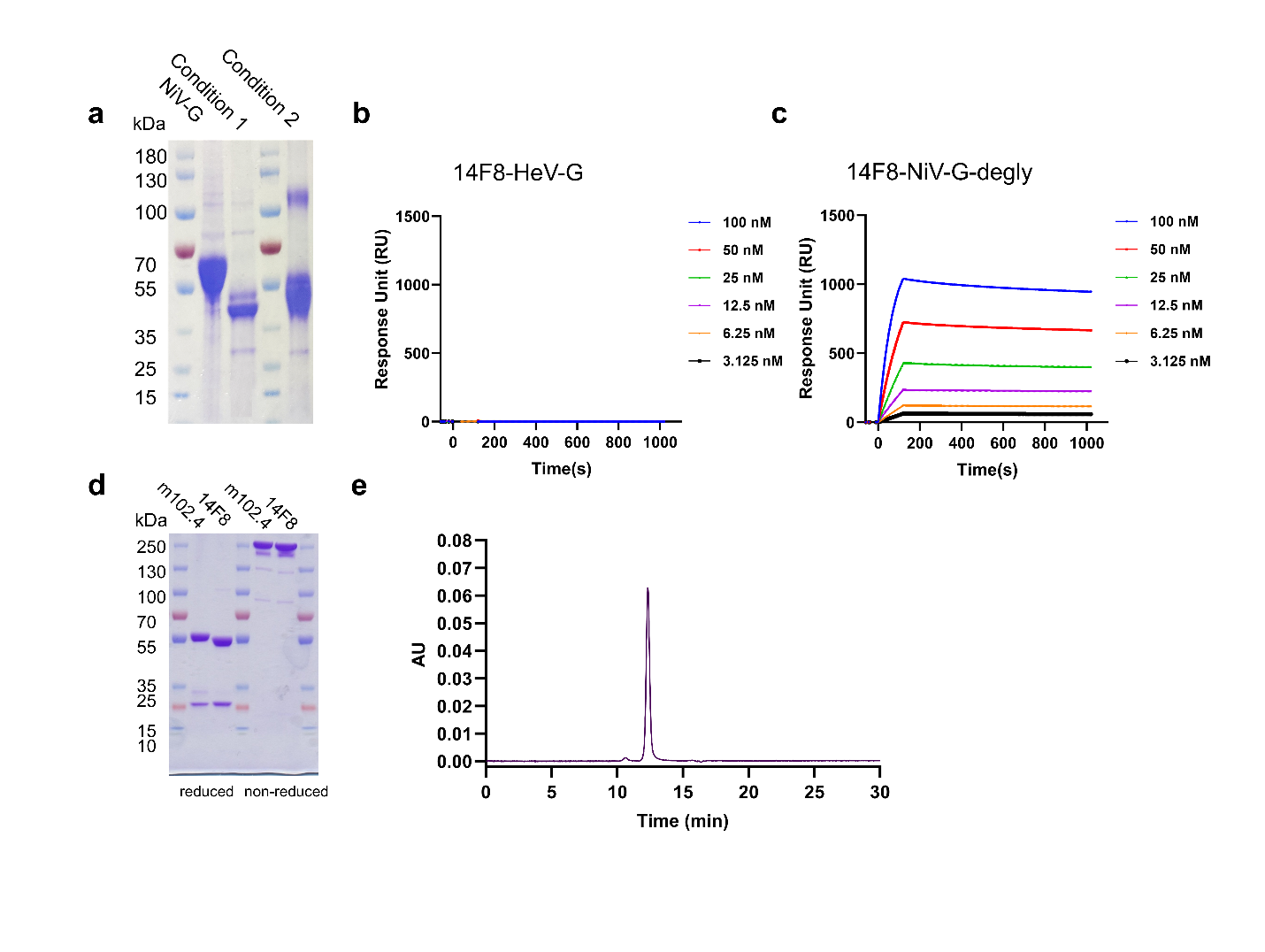


Expression and binding curves of G proteins. (a) NiV-G is deglycosylated under denaturation (condition1) or non-denaturation conditions (condition 2), the first lane represents untreated NiV-G used as a control. (b) Affinity curve of 14F8 with HeV-G protein. (c) Affinity curve of 14F8 with NiV-G protein in deglycosylated condition. (d) SDS-PAGE of antibody m102.4 and 14F8 (e) Size exclusion chromatography of 14F8.

**Figure S2.**
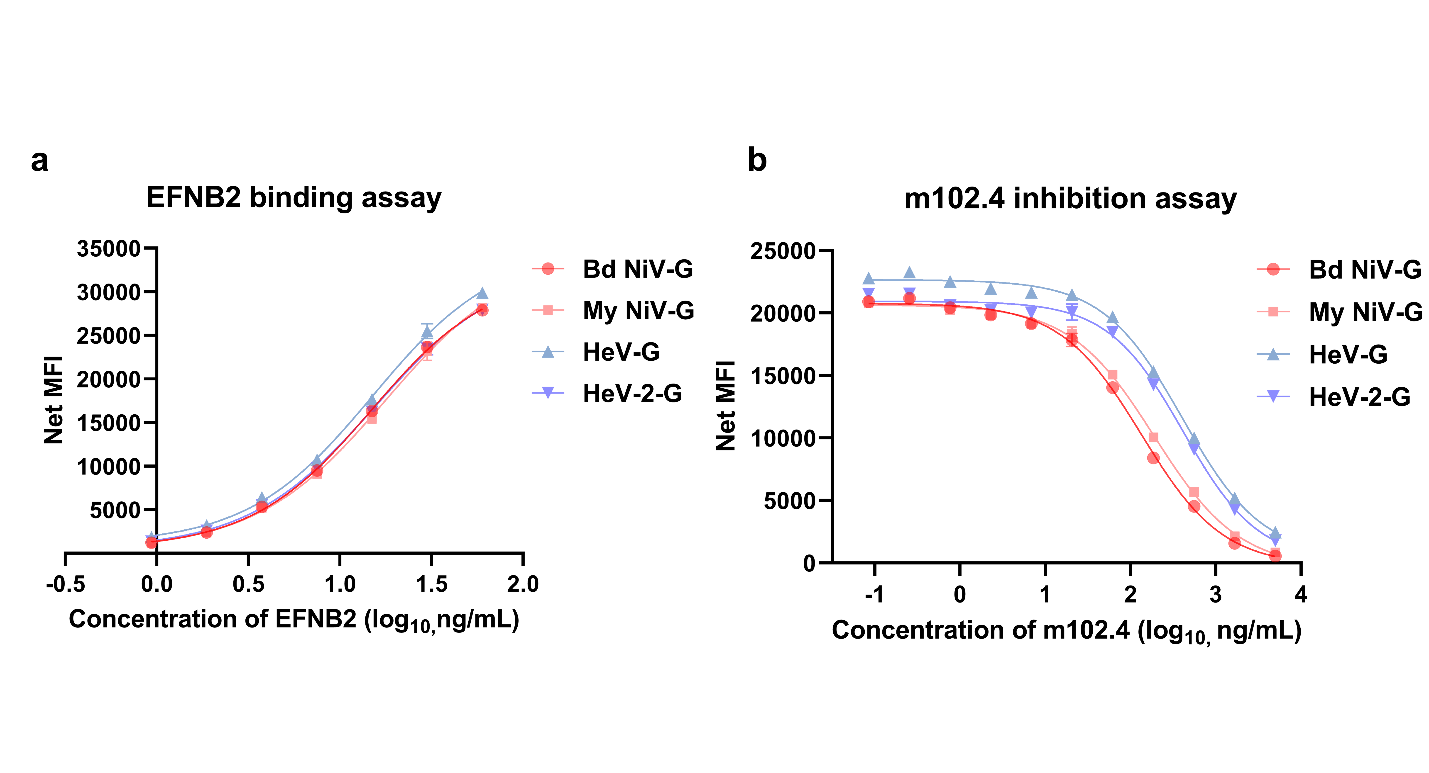


EFNB2 binding assay and m102.4 inhibition assay based on the Luminex platform. (a) Binding curve of G proteins coated Luminex beads with cell receptors ephrin B2. (b) Inhibition curves of m102.4.

**Figure S3.**
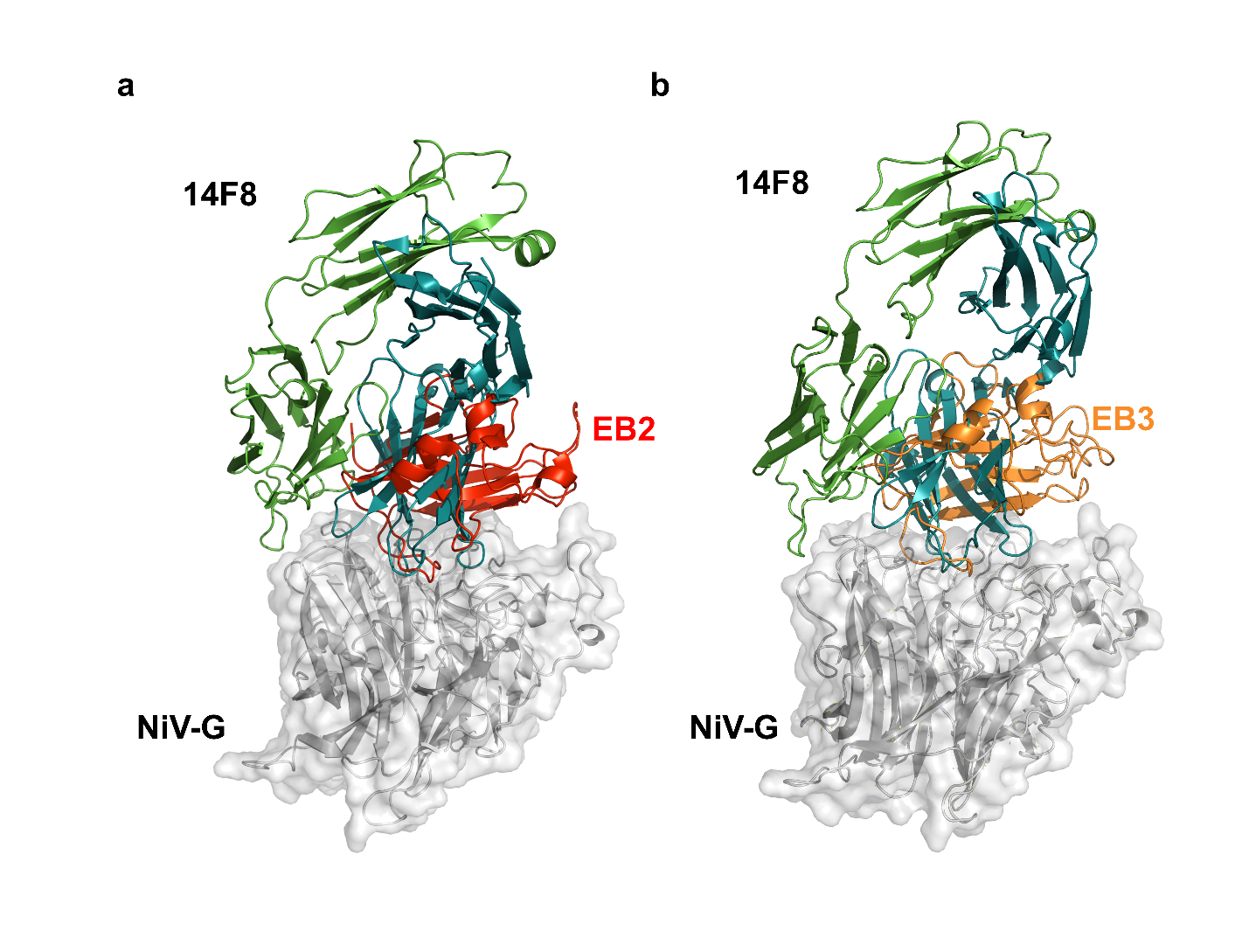


Alignment of protein complexes. Alignment of Nipah Virus attachment glycoprotein (G) in complex with neutralizing antibody 14F8 (a) and G protein in complex with Ephrin B2\B3 cell receptors (b) (PDB ID:2VSM,3D12).

**Figure S4.
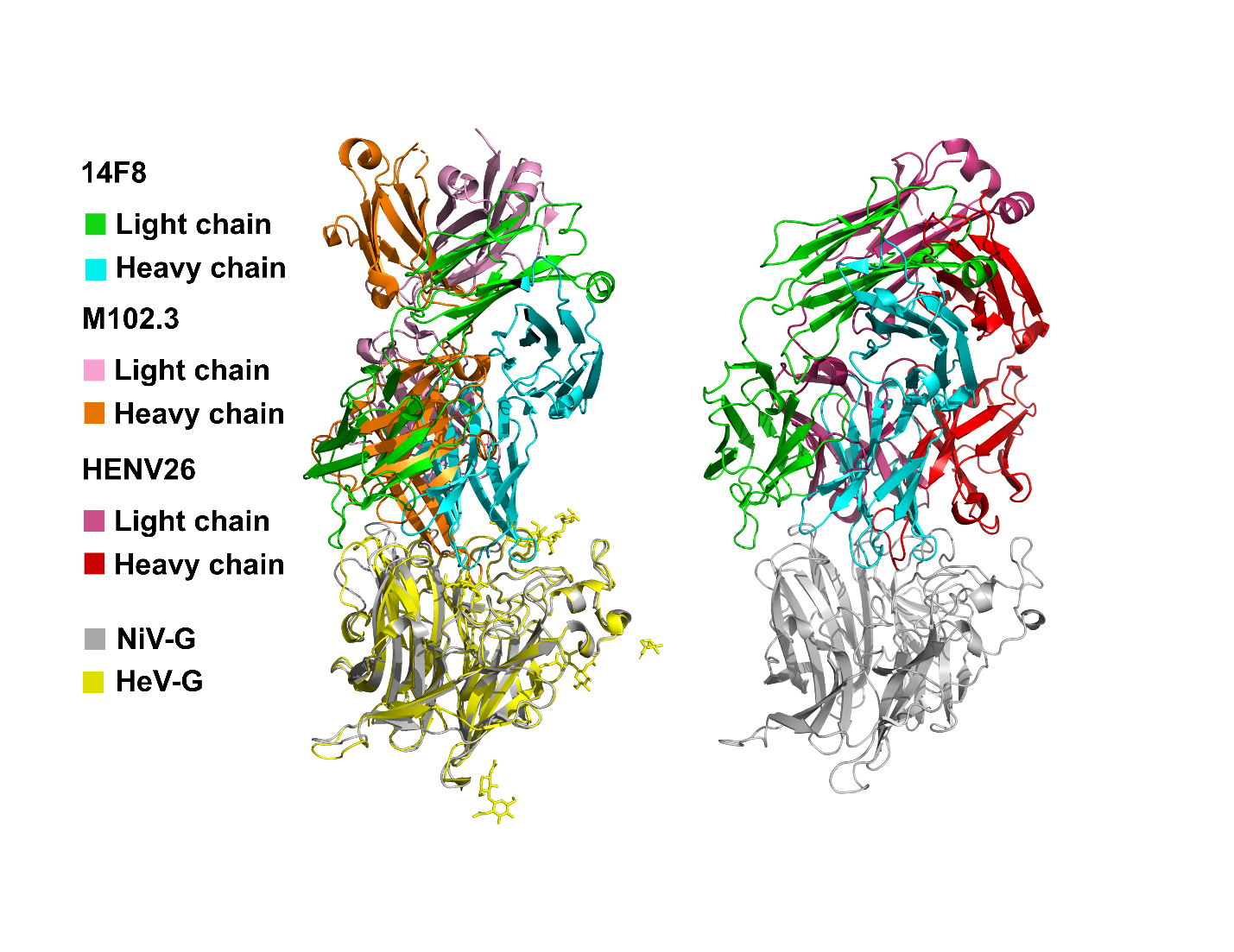
**

Alignment of antibody-antigen complexes. Alignment of Nipah Virus attachment glycoprotein (G) in complex with neutralizing antibody 14F8 and Hendra Virus G protein in complex with antibody m102.3 (PDB ID:6CMI) (left); alignment of Nipah Virus G protein in complex with neutralizing antibody 14F8 and Nipah Virus G protein in complex with antibody HENV-26 (PDB ID:6VY5).

**Figure S5.**


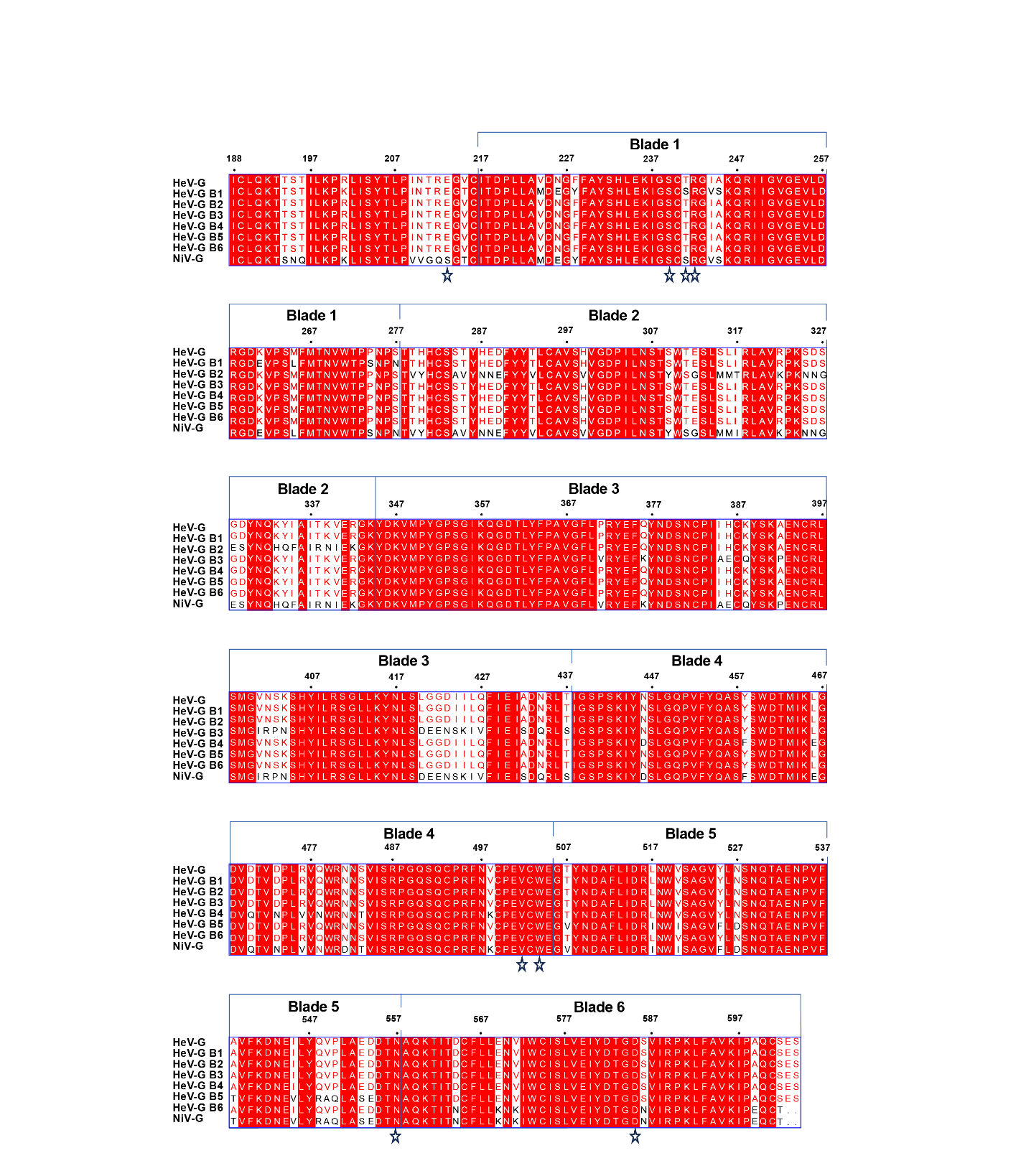


Amino acid sequence alignment of HeV-G mutants with NiV-G (Bangladesh strain) and HeV-G (GenBank: AF017149.3). The pentagram represents amino acids that interact with 14F8.

**Figure S6.**

**
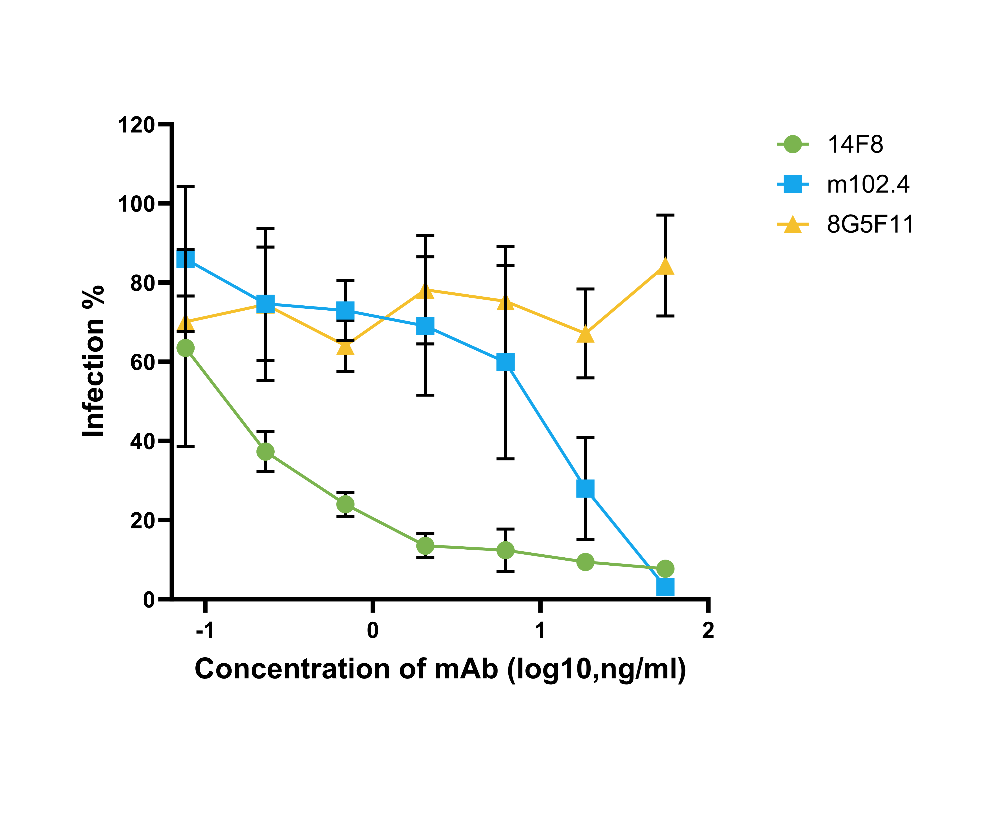
**

Comparison of the neutralization activity of 14F8, m102.4 and a non-specific control antibody (anti VSV-G, 8G5F11) against G-S586N HeV pseudoviruses using Ephrin B2-expressing 293T cells. Each point represents three technical replicates, and error bars represent SD.

**Figure S7.
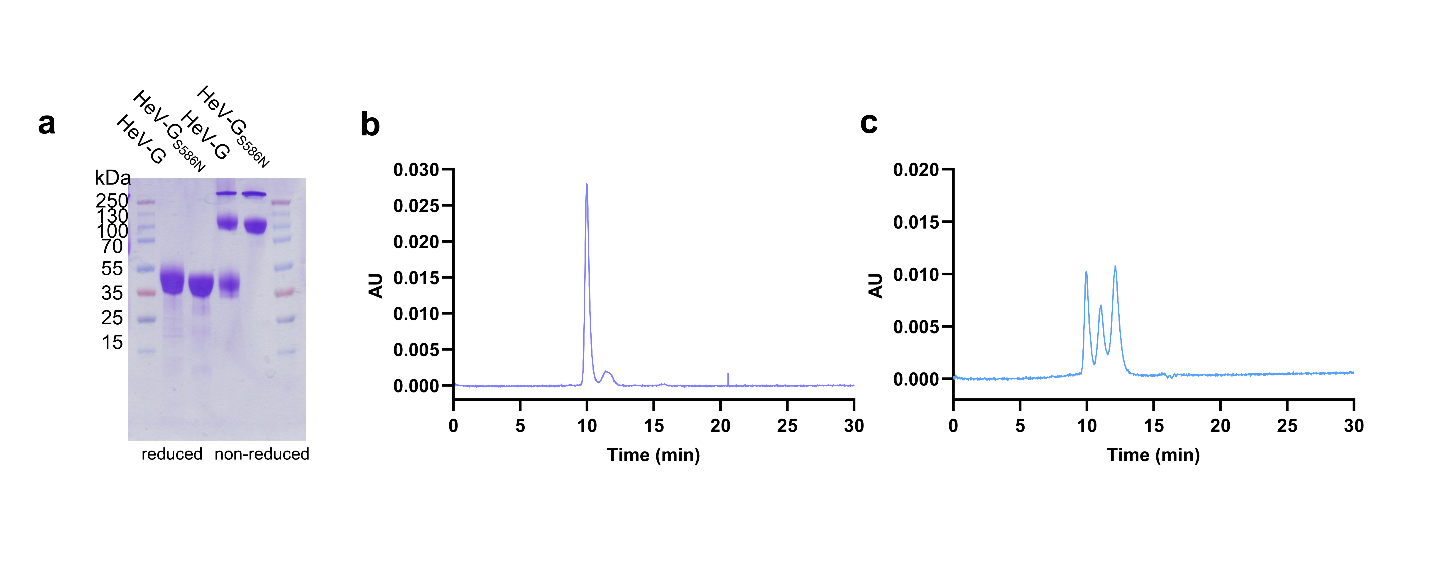
**

Characterization of HeV-G and HeV-GS586N proteins. (a) SDS-PAGE of purified HeV-G and HeV-G_S586N_. Size exclusion chromatography of purified HeV-G (b) and HeV-G_S586N_ (c).

**Figure S8.
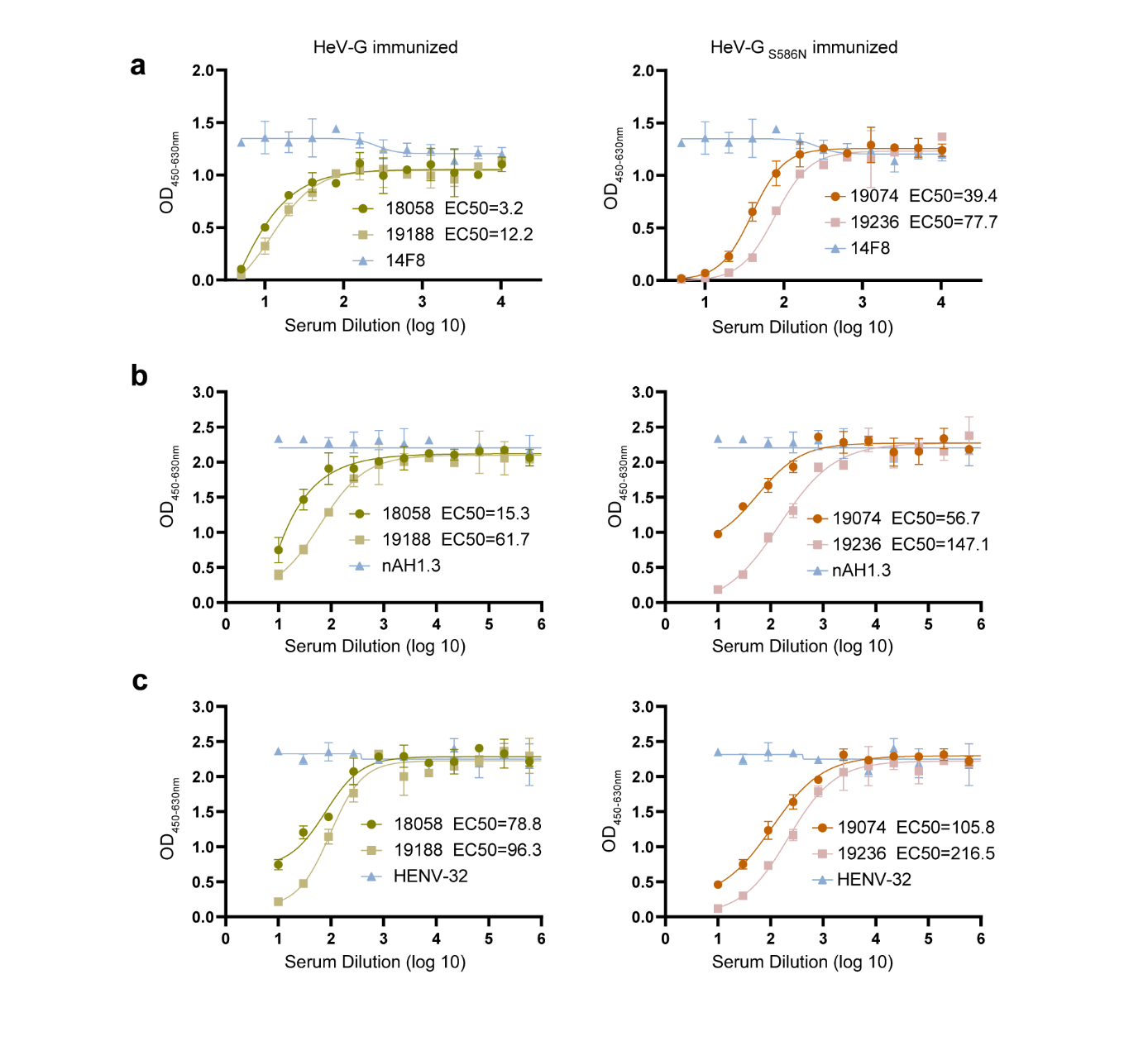
**

Serum competition ELISA against NiV-G. Purified NiV-G was coated. After blocking with PBS containing 2% BSA, serial dilutions of monkey sera were added and incubated, biotinylated IgG antibodies 14F8, nAH1.3, or HENV-32 were added, followed by HRP-conjugated streptavidin colorimetric reaction, and the absorbance was measured at 450–630 nm. The serum competition curves for 14F8(a), nAH1.3(b), and HENV-32(c) are presented, with each point representing the average of two technical replicates and error bars represent SD. The blue curves represent the control group, in which only the neutralizing antibody was added, without any serum. The left and right panels in each figure are from the same experiment and share the same control group.

**Figure S9.
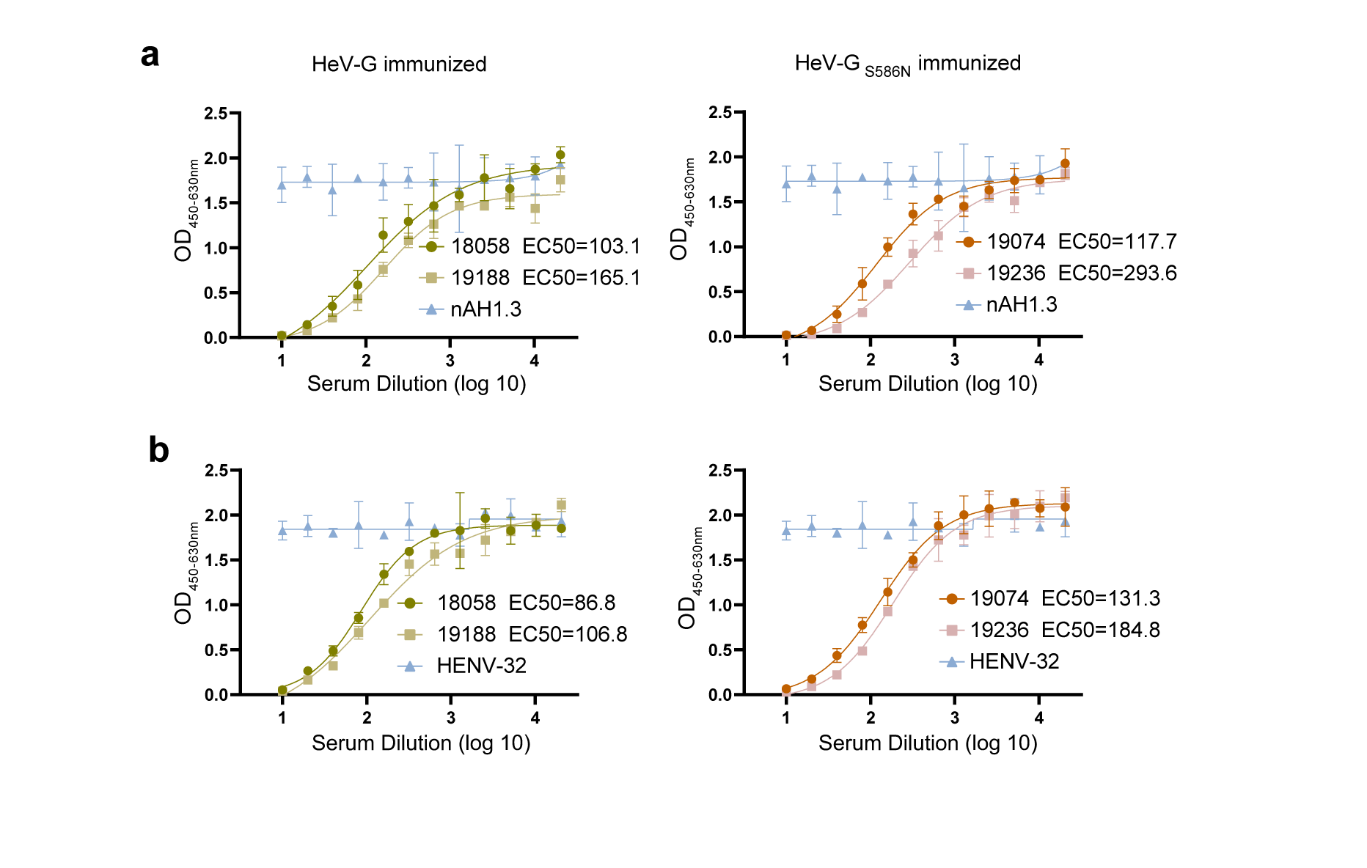
**

Serum competition ELISA against HeV-G. Purified HeV-G was coated. After blocking with PBS containing 2% BSA, serial dilutions of monkey sera were added and incubated, biotinylated IgG antibodies nAH1.3, or HENV-32 were added, followed by HRP-conjugated streptavidin colorimetric reaction, and the absorbance was measured at 450–630 nm. The serum competition curves for nAH1.3(a) and HENV-32(b) are presented, with each point representing the average of two technical replicates and error bars represent SD. The blue curves represent the control group, in which only the neutralizing antibody was added, without any serum. The left and right panels in each figure are from the same experiment and share the same control group.

**Figure S10.**


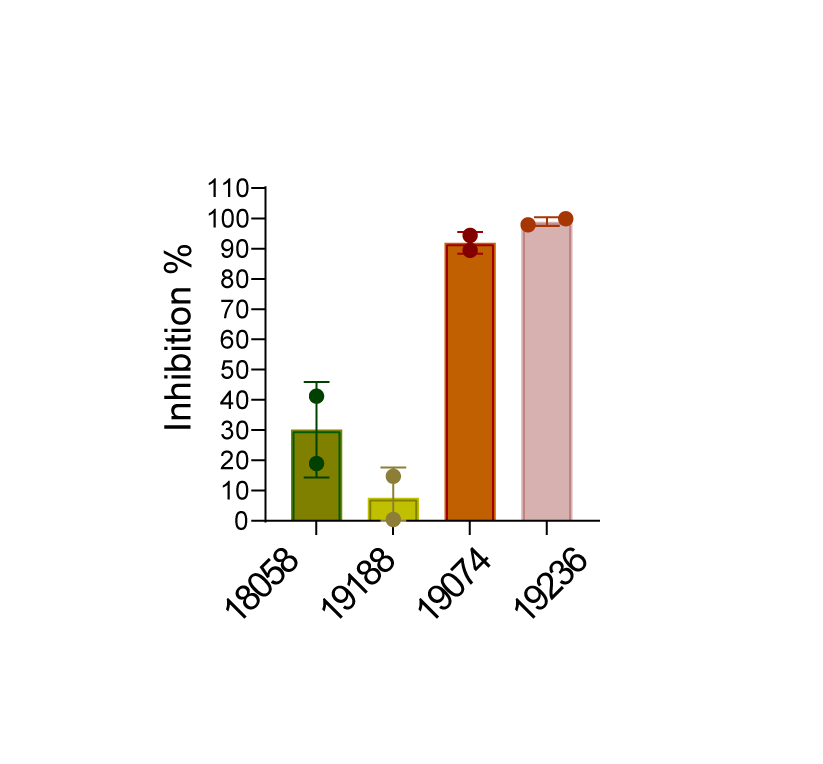


NiV neutralization assay with depleted monkey Sera. Monkey sera were added in duplicate to 96-well plates, followed by the addition of HeV-G (2 μg/well) and incubation at 37°C for 30 minutes. NiV pseudovirus was then added and incubated for 1 hour, after which 293T cells were added, the plates were incubated for 48 hours. The luciferase signal was measured. The figure shows the NiV pseudovirus inhibition rates after serum depletion from four monkeys, with each point representing one technical replicate and error bars represent SD. Monkeys 19074 and 19236 were immunized with HeV-G_S586N_, while monkeys 18058 and 19188 were immunized with wild-type HeV-G. The blank monkey serum was used as the negative control.

**Figure S11.
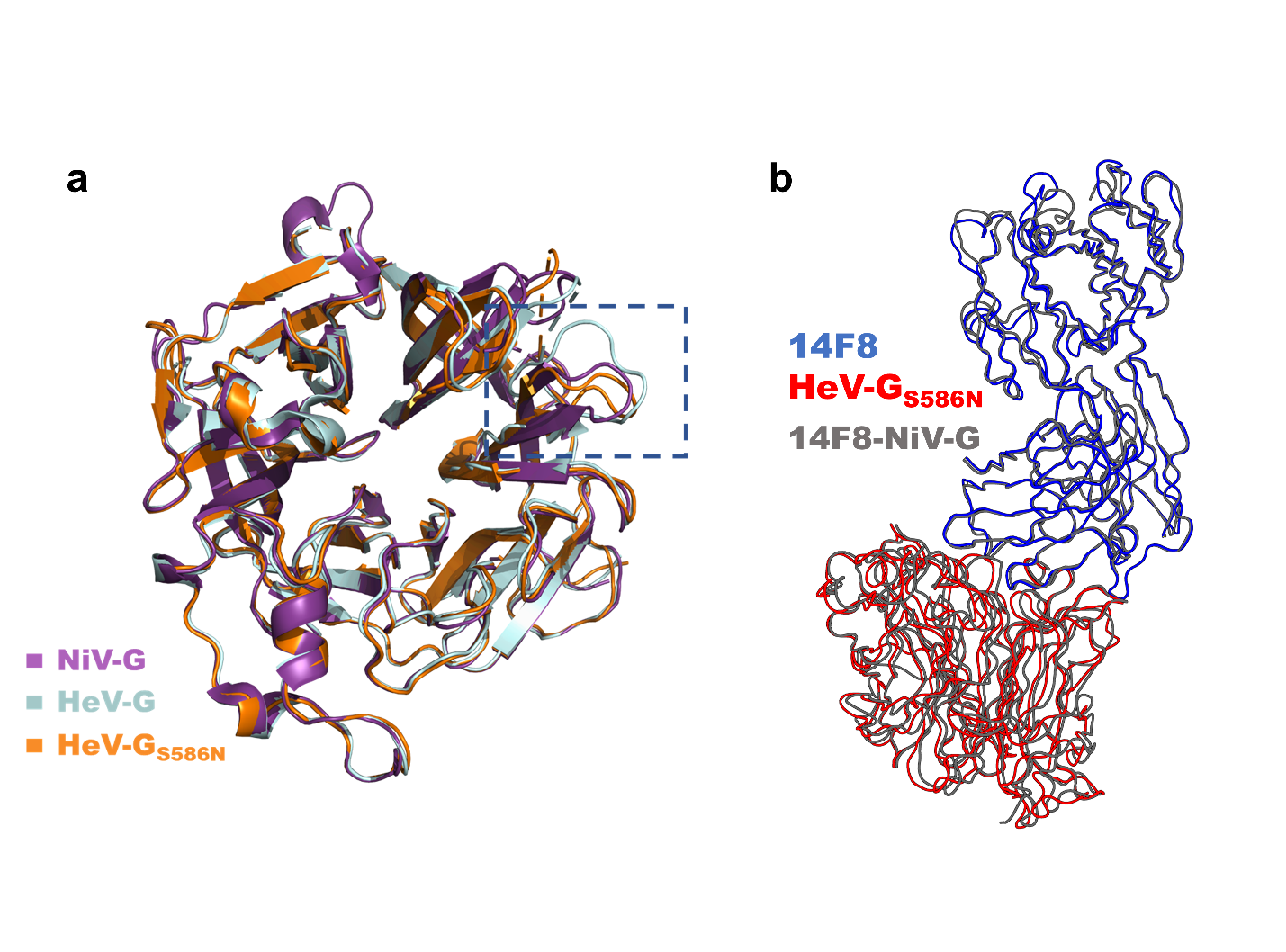
**

Alignment of proteins/complexes. (a) Alignment of HeV-G_S586N_, HeV-G (PDB ID:2VSK) and NiV-G (PDB ID:2VWD). The highlighted boxed region marks the area surrounding residue 586, where conformational rearrangements were observed. (b) Alignment of 14F8-NiV-G complex and 14F8-HeV-G_S586N_ complex.

**Table S1.**

Contact list between 14F8 with NiV-G

| Chain 1 | Residue | Chain 2 | Residue | Interaction type |
| --- | --- | --- | --- | --- |
| 14F8 light chain | His31.NE2 (CDRL1) | NiV-G | Asp585.O | Hydrogen bond interaction |
| 14F8 light chain | Ser32.OG  (CDRL1) | NiV-G | Asp585.OD1 | Hydrogen bond interaction |
| 14F8 light chain | Asn33.ND2  (CDRL1) | NiV-G | Gly214.O | Hydrogen bond interaction |
| 14F8 heavy chain | Asp100.OD1 (CDRH3) | NiV-G | Ser239.N | Hydrogen bond interaction |
| 14F8 heavy chain | Asp33.OD2  (CDRH1) | NiV-G | Ser241.OG | Hydrogen bond interaction |
| 14F8 heavy chain | Ser31.O  (CDRH1) | NiV-G | Arg242.NH1 | Hydrogen bond interaction |
| 14F8 heavy chain | Asn73.ND2  (FRH3) | NiV-G | Trp504.O | Hydrogen bond interaction |
| 14F8 heavy chain | [Lys75.NZ](https://lys75.nz/#tdsub)  (FRH3) | NiV-G | Ile502.O | Hydrogen bond interaction |
| 14F8 heavy chain | Thr57.O  (CDRH2) | NiV-G | Asn557.ND2 | Hydrogen bond interaction |
| 14F8 heavy chain | [Lys75.NZ](https://lys75.nz/#tdsub)  (FRH3) | NiV-G | Glu501.OE2 | Salt bridge |

**Table S2.**

Contact list between 14F8 with HeV-G_S586N_

| Chain 1 | Residue | Chain 2 | Residue | Interaction type |
| --- | --- | --- | --- | --- |
| 14F8 light chain | His31.NE2  (CDRL1) | HeV-G_S586N_ | Asp585.O | Hydrogen bond interaction |
| 14F8 light chain | Ser32.OG  (CDRL1) | HeV-G_S586N_ | Asp585.OD1 | Hydrogen bond interaction |
| 14F8 light chain | Asn33.N  (CDRL1) | HeV-G_S586N_ | Gly214.N | Hydrogen bond interaction |
| 14F8 heavy chain | [Asp100.CA](https://asp100.ca/#tdsub)  (CDRH3) | HeV-G_S586N_ | Ser239.OG | Hydrogen bond interaction |
| 14F8 heavy chain | Asp33.OD1  (CDRH1) | HeV-G_S586N_ | Thr241.OG1 | Hydrogen bond interaction |
| 14F8 heavy chain | Lys71.O  (FRH3) | HeV-G_S586N_ | [Gln490.CG](https://gln490.cg/#tdsub) | Hydrogen bond interaction |
| 14F8 heavy chain | Ser74.OG  (FRH3) | HeV-G_S586N_ | Val502.O | Hydrogen bond interaction |
| 14F8 heavy chain | Ser74.OG  (FRH3) | HeV-G_S586N_ | [Cys503.CA](https://cys503.ca/#tdsub) | Hydrogen bond interaction |
| 14F8 heavy chain | Asn73.ND2  (FRH3) | HeV-G_S586N_ | Glu505.OE1 | Hydrogen bond interaction |
